# Characterization of two novel mycoviruses from *Penicillium digitatum* and the related fungicide resistance analysis

**DOI:** 10.1038/s41598-018-23807-3

**Published:** 2018-04-03

**Authors:** Yuhui Niu, Yongze Yuan, Jiali Mao, Zhu Yang, Qianwen Cao, Tingfu Zhang, Shengqiang Wang, Deli Liu

**Affiliations:** Hubei Key Laboratory of Genetic Regulation and Integrative Biology, School of Life Sciences, Central China Normal University, Wuhan, 430079 P. R. China

## Abstract

Pathogenic fungi including *Penicillium digitatum* and *Penicillium italicum* are the main destructive pathogens in the citrus industry, causing great losses during postharvest process. To our knowledge, only one mycovirus from *P. digitatum* has been reported, and the prevalence of such mycoviruses against citrus postharvest pathogenic fungi and their genotyping were still under investigation. In the present study, we showed that 39 of 152 *Penicillium* isolates from main citrus-growing areas in China were infected with various mycoviruses belonging to polymycoviruses, Narna-like viruses, and families *Totiviridae*, *Partitivirdae* and *Chrysoviridae*. The next generation sequencing (NGS) towards virus genome library and the following molecular analysis revealed two novel mycoviruses *Penicillium digitatum* polymycovirus 1 (PdPmV1) and *Penicillium digitatum* Narna-like virus 1 (PdNLV1), coexisting in *P. digitatum* strain HS-RH2. The fungicide-resistant *P. digitatum* strains HS-F6 and HS-E9 coinfected by PdPmV1 and PdNLV1 exhibited obvious reduction in triazole drug prochloraz resistance by mycelial growth analysis on both PDA plates and citrus fruit epidermis with given prochloraz concentration. This report at the first time characterized two novel mycoviruses from *P. digitatum* and revealed the mycovirus-induced reduction of fungicide resistance.

## Introduction

Citrus fruit is one of valuable crops in agricultural fruit markets. A quantity of harvested citrus fruits decay by *P. digitatum*, *P. italicum*, *P. crustosum*, and other filamentous fungi during postharvest handling^1,2^. Currently, synthetic chemicals including triazole drugs have been predominantly applied to control these phytopathogens^3–6^. However, the application of such chemicals is becoming increasingly problematic due to the emergence of fungicide-resistant strains and the drug-induced toxicological risks to public health^4,6–8^. Thus, there is an urgent need to develop alternative method(s) like mycovirus-related biocontrol against these postharvest diseases.

Mycoviruses have been described in all major group of fungi. The reported mycoviruses are mainly double-stranded (ds) RNA mycoviruses or single-stranded (ss) RNA mycoviruses. The dsRNA mycoviruses are classified into seven families including *Totiviridae*, *Partitiviridae* and *Chrysoviridae*, and the ssRNA mycoviruses are classified into six families including *Narnaviridae*^9^.

Concerning RNA mycoviruses sharing characteristics between dsRNA and positive-strand ssRNA viruses, a novel family ‘Polymycoviridae’ has been proposed from *Beauveria bassiana* virus screening but not accepted by current International Committee on Taxonomy of Viruses (ICTV)^10^. This newly proposed family mainly comprised non-conventionally encapsidated mycoviruses (polymycoviruses) with four to seven dsRNA segments (0.9–2.4 kbp in length) as genome structure^10,11^. According to the updated information on polymycovirus molecular biology, four largest dsRNAs (dsRNA1–4), encoding functional proteins, were found in almost all polymycoviruses as their essential genome components, while the remaining dsRNA segments were not uniformly identified concerning their function(s) in the mycovirus physiology. For details, dsRNA1 and dsRNA3 have been speculated to encode RNA-dependent RNA polymerase (RdRp) and methyltransferase respectively, and dsRNA2 and dsRNA4 encode proteins with specific motifs, cysteine-rich, zinc finger-like and arginine repeats (R-R, R-X-R, R-R-R) for the former and proline-alanine-serine (PAS) rich for the latter^10–12^. The arginine repeats in dsRNA2-encoding protein have been considered as endoplasmic reticulum (ER) retention signals, normally found in transmembrane proteins and functioning in virus replication^10^. The proline-alanine-serine (PAS) rich protein encoded by dsRNA4 was identified to coat viral dsRNA in an unconventional way^10^.

As ssRNA mycoviruses with the simplest genomes, the family *Narnaviridae* accommodates members each containing a single linear (+)ssRNA molecule, uncapsidated, 2.3–3.6 kb in length and encompassing a single ORF that encodes the RdRp^9,13^. According to subcellular location, the members of family *Narnaviridae* have been classified into two genera *Narnavirus* and *Mitovirus*, the former confined to cytosol and conventionally found in the yeast *Saccharomyces cerevisiae* and *Phytophthora infestans*, and the latter localized to mitochondria and only reported in filamentous fungi^9,13–15^. Up to date, the extensive evidence on dsRNA and/or ssRNA mycoviruses has been addressed in fungal phytopathogens including *Cryphonectria parasitica*, *Sclerotinia sclerotiorum*, *Rosellinia necatrix*, *Botrytis* species, and *Fusarium* species^16^. In contrast, apart from *Penicillium digitatum* virus 1 (PdV1)^17^, no other mycovirus has been reported from citrus pathogenic fungi (*Penicillium* species).

As previously reported, mycoviruses usually have little effects on their hosts with several exceptions. Mycoviruses such as *Cryphonectria* hypovirus 1 have been identified to cause hypovirulence in their host fungi^18^. Additionally, the infection of dsRNA viruses to ascomycetous yeast *Saccharomyce scerevisiae* enables the host to secrete protein toxins to favor its growth^19^. The phenotypic response(s) to mycoviruses infection(s) are of interest to gain more knowledge on virus-host interactions.

In this study, we evaluated the presence of mycovirus in citrus pathogenic fungi (*Penicillium* species) through a total of 152 isolates from main citrus-growing provinces in China. From one of all 39 mycovirus-infected *Penicillium* strains, we identified and characterized two novel mycoviruses belonging to Narna-like viruses and provisionally designated family ‘Polymycoviridae’, and further assessed host phenotypic response(s) to the infection of these identified mycoviruses under triazole drug prochloraz conditions.

## Results

### Screening of dsRNA elements in citrus postharvest pathogenic fungi

To evaluate the prevalence of dsRNA elements in citrus pathogenic fungi (*Penicillium* species), we isolated 152 strains from decayed citrus fruit epidermis collected from main citrus-growing provinces in China including Hubei, Sichuan, Jiangxi and Yunnan, and the results were summarized in Table S1. The dsRNA elements, insusceptible to DNase I and to S1 nuclease, were separated by agarose gel electrophoresis from thirty-nine strains in that 1) thirty-five strains of 89 *P. digitatum* isolates and 2) three *P. crustosum* and one *P. italicum* strains of the rest 63 *Penicillium* isolates. Based on the dsRNA banding pattern (Table S1), all the 39 *Penicillium* strains were classified into five groups (group1–5) with their representative electrophoretic profiles (Fig. S1). Group1, including three *P. crustosum* isolates (SC-12, SC-19 and SC-20) and *P. italicum* isolate YN-1, consisted of 4 dsRNA segments between 2.9–4 kbp in length (Fig. S1A), each sharing high sequence similarity with *Penicillium chrysogenum* virus (PcV, identity >85%). Group2–5 represented *P. digitatum* strains with diverse dsRNA banding patterns. Group2 including 24 isolates harbored a monosegmented ~5.2 kbp dsRNA (Fig. S1B), sharing high sequence similarity with PdV1, the typical member of *Victorivirus*. Group3 including 9 isolates harbored two dsRNA segments with ~2.0 kbp and ~1.8 kbp in length (Fig. S1C), each sharing high sequence similarity with *Penicillium stoloniferum* Virus S (PsV-S, identity >90%). Group4, corresponding to only one strain (HB-22), consisted of three dsRNAs with size of ~5.2 kbp, ~2.0 kbp and ~1.8 kbp (Fig. S1D), the largest sharing high sequence similarity with PdV1 and the rest two sharing high sequence similarity with PsV-S. Group5, also corresponding to only one strain (HB-36), consisted of six dsRNAs (dsRNAs 1 to 6) with ~5.2 kbp, ~2.3 kbp, ~2.3 kbp, ~2.0 kbp, ~1.6 kbp, and ~1.3 kbp in size (Fig. S1E), dsRNA1 sharing high sequence similarity with PdV1 except several nucleotides variance, thus labeled as PdV1 dsRNA, and dsRNA2-6, with lower sequence similarity to reported mycoviruses, constituting the genome of two mycoviruses belonging to Narna-like (NL) viruses and newly proposed family ‘Polymycoviridae’ (Pm), thus labeled as NL and Pm dsRNAs. Considering that members of either Narna-like viruses or polymycoviruses were seldom reported in *Penicillium* strains, these two mycoviruses harboring dsRNA2-6 were subjected to further analysis in the following study. To this end, an isolate (named HS-RH2), containing the two putatively novel mycoviruses, was prepared through curing the dsRNA1 virus (PdV1), and the lacking of target dsRNA segment was verified by northern blot (Fig. S2).

### Characterization of a novel polymycovirus from *P. digitatum*

**Figure 1:**
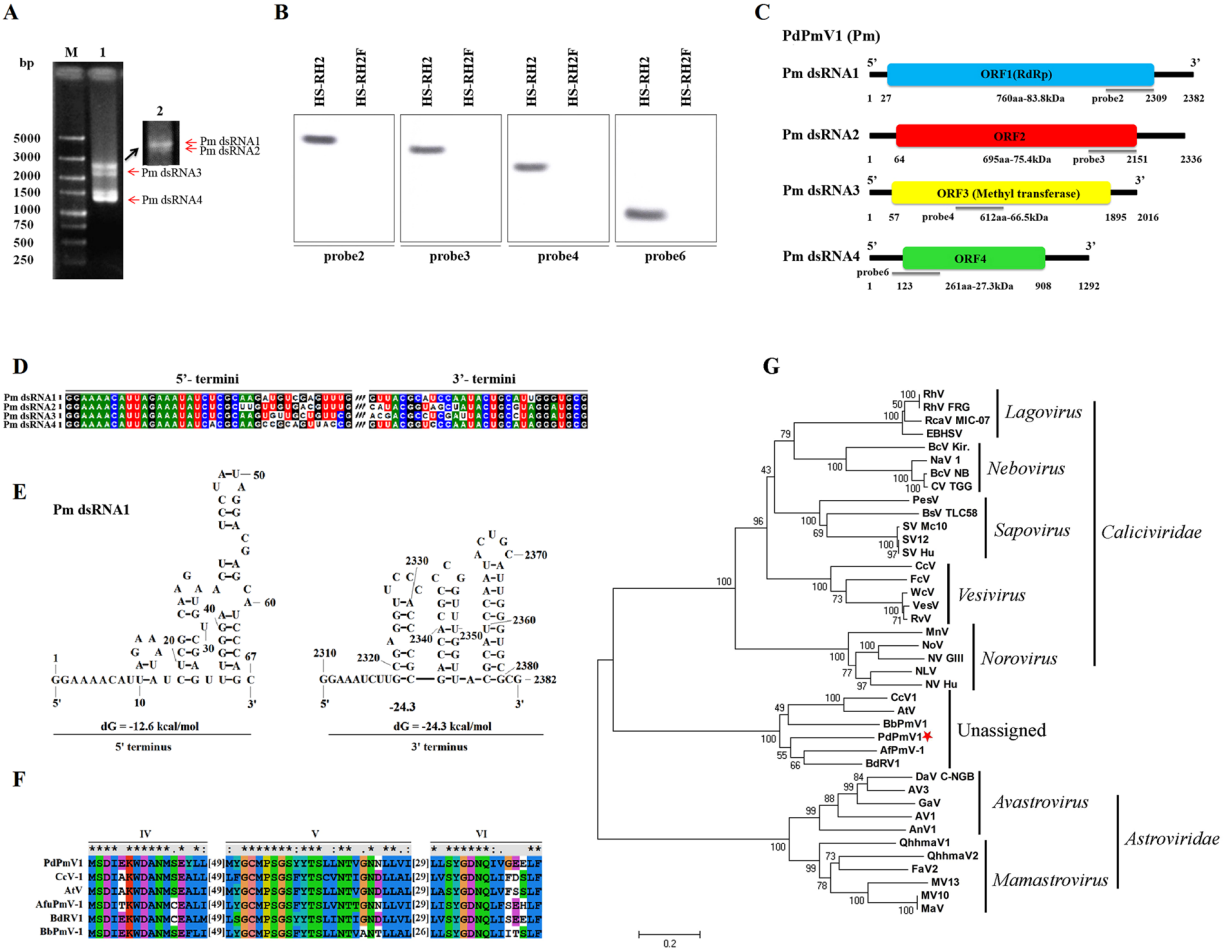
Molecular characterization and evolutionary analysis of PdPmV1. (**A**) Electrophoretic profile on a 1% agarose gel of dsRNA preparations extracted from *P. digitatum* isolate HS-RH2 after digestion with DNase I and S1 nuclease, and stained with ethidium bromide (lane 1). PdPmV1 (Pm) dsRNA1 and dsRNA2 were separated through 2% agarose gel electrophoresis (lane 2 and the corresponding full gel image was shown in Fig. S5). The Pm dsRNA1 to 4 were indicated by red arrow. Lane M, DNA size marker (DS 5000, TaKaRa, Dalian, China). (**B**) Northern blot analysis of PdPmV1 dsRNAs in HS-RH2 and HS-RH2F. The dsRNAs were separated under denaturing electrophoresis conditions, blotted onto nylon membranes, and probed by DIG-labeled DNA fragments (Fig. S6). The position of the probes (probe 2, 3, 4 and 6 to blot Pm dsRNA1 to 4 respectively) was shown in panel C. (**C**) Schematic representation of the genetic organization of PdPmV1. (**D**) Conserved sequences of the 5′-termini and 3′-termini of the dsRNAs of PdPmV1. (**E**) Secondary structures proposed for the terminal regions of Pm dsRNA1 with the lowest energy. (**F**) Comparison of the conserved motifs of RdRps encoded by PdPmV1 and other selected mycoviruses. The alignment was performed by the program CLUSTAL_X with manual modification. Three conserved RdRp motifs corresponding to motifs IV, V, and VI were shown. The asterisks signified identical amino acid residues, colons signified highly conserved residues, and single dots signified less conserved but related residues. Numbers within the brackets indicated the number of aa not shown. (**G**) Phylogenetic analysis of PdPmV1 RdRp and other selected RdRps. Members of the families *Astroviridae*, *Caliciviridae* and the proposed *Polymycoviridae* listed in Table S2 were selected, and the phylogenetic tree for RdRP sequences was constructed using the neighbor-joining method of MEGA software (version 6.0) with bootstrapping analysis of 1000 replicates.

The dsRNA2-6 isolated from HS-RH2 was purified and used for cDNA library preparation and subsequent next generation sequencing (NGS) using Illumina platform. The obtained contigs were subjected to BLAST search in NCBI databases to identify viral sequences. The complete nucleotide sequences of these dsRNAs were finally obtained by contig assembling. BLAST analysis revealed that four dsRNAs (dsRNA2-4, 6) constituted the genome of a novel polymycovirus (Fig. 1A,B), tentatively designated *Penicillium digitatum* polymycovirus 1 (PdPmV1) in the present study. For this reason, dsRNA2, 3, 4 and 6, labeled in order of increasing agrose-gel electrophoresis mobility (Fig. S1E), were referred to as Pm dsRNA1 to 4 (Fig. 1A). A schematic representation of the genetic organization of PdPmV1 is shown in Fig. 1C. The entire lengths of each dsRNA are 2382, 2336, 2016 and 1292 bp, and these dsRNAs respectively contain a single ORF on the plus-strand putatively encoding proteins 760 aa (83.8 kDa), 695aa (75.4 kDa), 612 (66.5 kDa) and 261aa (27.3 kDa) in size, flanked by 5′- and 3′- untranslated regions (UTRs). The 5′-UTRs of the four segments are 26, 63, 56 and 122 bp in size, while the 3′-UTRs are 73, 185, 121 and 384 bp in size. The 5′- and 3′-termini of coding strands of all four dsRNAs respectively contained conserved 19nt sequence (GGAAAACAUUAGAAAUAUC) and 6nt sequence (UGCGCG) (Fig. 1D), and were predicted to fold into stable stem-loop structures, as illustrated for Pm dsRNA1 (Fig. 1E). The full-length genomic cDNA sequences of Pm dsRNA1 to 4 were deposited in the GenBank database with accession numbers MF317878-MF317881.

A homology analysis revealed that the protein encoded by Pm dsRNA1 is most closely related to RdRp encoded by *Aspergillus fumigatus* tetramycovirus-1 (AfuPmV-1) by sharing 48% aa sequence identity (Accession no. CDP74618, E value = 0.00), and also similar to the RdRp of *Botryosphaeria dothidea* virus 1 (BdRV1, accession no. ALZ41794, E value = 4e-175 and 46% identity) and putative RdRp of *Cladosporium cladosporioides* virus 1 (CcV1, accession no. YP_009052470, E value = 7e-166 and 39% identity). The deduced aa sequence of the Pm dsRNA1 ORF contained three conserved motifs (IV-VI) found in the picorna-like RdRp family of positive-strand, RNA eukaryotic viruses (RdRp_1, pfam00680) (Fig. 1F). Concerning motif VI, the GDD motif in RdRp is replaced with a GDNQ motif, as described by Kotta-Loizou^10^. A phylogenetic tree generated based on an alignment of RdRp sequences (listed in Table S2) places PdPmV1 into the clade of a newly proposed family ‘Polymycoviridae’ (but not assigned by ICTV yet) (Fig. 1G).

The Pm dsRNA2 encoded a hypothetical protein, rich in arginine repeats (R-R, R-X-R, R-R-R), containing a cysteine-rich, zinc finger-like motif (Fig. S3A), and sharing low similarity with other hypothetical proteins encoded by dsRNA2 of AfuPmV-1 (accession no. CDP74619, E value = 3e-114 and 36% identity) and dsRNA2 of BdRV1 (accession no. YP_009342447, E value = 2e-98 and 34% identity).

The deduced aa sequence of protein encoded by Pm dsRNA3 shared the highest identity of 40% with a methyltransferase encoded by dsRNA3 of AfuPmV-1 (Accession no. CDP74620, and E value = 6e-131) and low similarity with unassigned proteins encoded by dsRNA of *Melampsora lini* (Accession no. CAA45724; E value = 2e-128 and 39% identity) and BdRV1 (Accession no. AIZ41796; E value = 1e-104 and 36% identity). Additionally, a conserved protease domain of *S*-adenosylmethionine-dependent methyltransferases (cd02440 and E value = 4.39e-03) was observed in the N-terminal region (positions 132–242) of the present Pm dsRNA-encoding protein, and a conserved catalytic motif of methyltransferase was also detected in its primary structure (Fig. S3B).

The Pm dsRNA4 encoded a Proline-Alanine-Serine rich protein (PASrp), containing 8.04% proline, 12.26% alanine and 6.90% serine residues out of total 261 aa and sharing 35% aa sequence identity with a hypothetical protein encoded by dsRNA4 of BdRV1 (accession no. YP_009342471; E value = 3e-30), a hypothetical protein of CcV1 (Accession no. YP_009052473; E value = 8e-12) and a PASrp of *Beauveria bassiana* polymycovirus 1 (BbPmV1, accession no. YP_009352878; E value = 4e-11).

### Characterization of a novel Narna-like virus from *P. digitatum*

**Figure 2:**
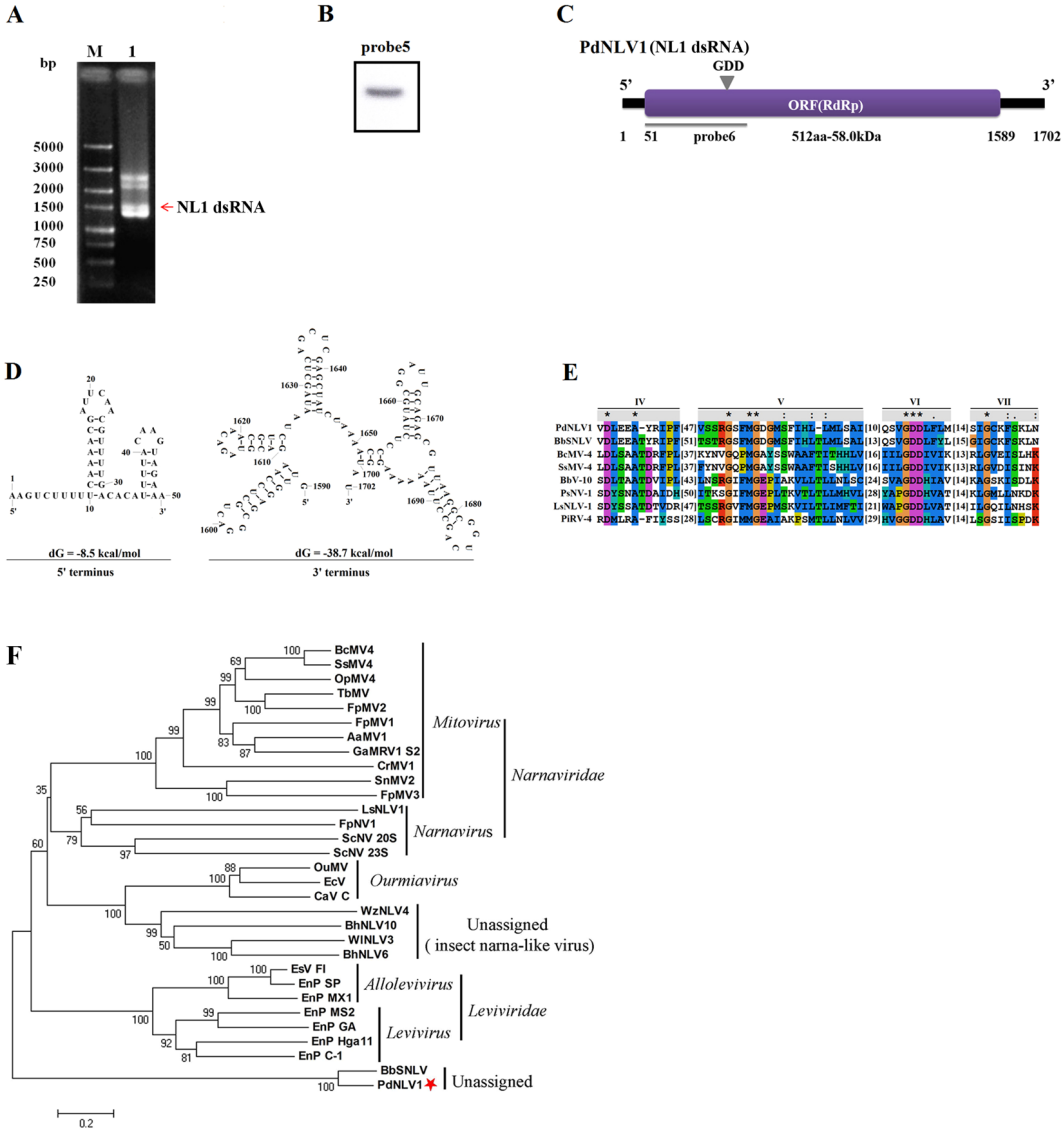
Molecular characterization and evolutionary analysis of PdNLV1. (**A**) Electrophoretic profile on a 1% agarose gel of dsRNA preparations extracted from *P. digitatum* isolate HS-RH2 after digestion with DNase I and S1 nuclease, and stained with ethidium bromide (lane 1). The dsRNA of PdNLV1 was indicated by red arrows. Lane M, DNA size marker (DS 5000, TaKaRa, Dalian, China). (**B**) Northern blot analysis of PdNLV1 dsRNA extracted from *P. digitatum* isolate HS-RH2 (Fig. S7). The position of the specific probe (probe 5) was shown in panel C. (**C**) Schematic representation of the genetic organization of PdNLV1. (**D**) Predicted secondary structure of the PdNLV1 5′- and 3′-UTRs with the lowest energy. (**E**) Comparison of the conserved motifs of RdRps encoded by PdNLV1 and other selected mycoviruses. Four conserved RdRp motifs corresponding to motifs IV, V, VI and VII were shown. The asterisks signified identical amino acid residues, colons signified highly conserved residues, and single dots signified less conserved but related residues. Numbers within the brackets indicated the number of aa not shown. (**F**) Phylogenetic analysis of PdNLV1 RdRp and other selected RdRps. Members of narnaviruses and Narna-like viruses in databases (Table S2) were selected, and the phylogenetic trees for RdRp sequences were constructed using the neighbor-joining method of MEGA version 6 software with bootstrapping analysis of 1000 replicates.

Besides the four dsRNAs of PdPmV1, HS-RH2 also harbors a ~1600bp dsRNA segment which is labeled as NL1 dsRNA according to the following molecular characterization (Fig. 2A,B). Sequencing of this dsRNA element indicated a 1,702 bp full-length, encoding a 512 aa (58 kDa) polypeptide and flanked by a 50 bp 5′-UTR and a 113 bp 3′-UTR (Fig. 2C). Secondary structure analysis of the positive strand using the mfold server predicted the formation of multiple stem-loops at both 3′- and 5′-UTR (Fig. 2D), which is in accordance with the characteristic of reported narnaviruses and Narna-like viruses. BLAST and multiple protein alignment analysis revealed that the polypeptide has great similarity with the RdRp of *Beauveria bassiana* small Narna-like virus (BbSNLV; E-value 0, 81% identity) and contains four motifs (IV-VII) of RdRps, including the conserved GDD motif (Fig. 2E). The virus was named *Penicillium digitatum* Narna-like virus 1 (PdNLV1), and the full-length genomic cDNA sequence of its dsRNA was deposited in the GenBank database with accession number MF322872. Phylogenetic analysis based on the selected RdRps (listed in Table S2) showed that PdNLV1 and BbSNLV were closely clustered as Narna-like viruses (Fig. 2F).

### Virus purification and peptide mass fingerprinting (PMF) analysis of viral protein

We exploited sucrose gradient ultracentrifugation to isolate virus and/or virus-like particles from HB-36 and HS-RH2. Under TEM scanning, we only observed regularly isometric virus-like particles from HB-36 rather than HS-RH2. These virus-like particles in ~33 nm diameter were further verified to be virions of the *Victorivirus* with dsRNA1 genome (i.e. the virus particles of previously reported PdV1)^17^ (Fig. S2C,D). Thus, we failed to obtain conventional virus-like particles from HS-RH2, the strain containing the newly identified mycoviruses in the present study. We also purified virus dsRNAs (Pm dsRNA1-4 and NL1 dsRNA) from HS-RH2 by previously reported methods^11,12^, however we still failed to observe any viral or viral like particles under TEM scanning. In the present work, the viral fraction concentrated by sucrose- or CsCl-gradient-ultracentrifugation was verified to contain all the PdPmV1 and PdNLV1 dsRNAs, and the further purified dsRNA mixture including naked Pm dsRNA1-4 and NL1 dsRNA displayed infectious ability to transfect *P. digitatum* recipients (e.g. HS-F6I and HS-E9I). These evidences ensured the existence of PdPmV1 and PdNLV1 in the TEM sample and thus confirmed no regular virus particles associated with the viruses from HS-RH2. On the other hand, regarding viral protein components, SDS-PAGE showed a major protein band with molecular mass of ~27 kDa (Fig. S2E), just corresponding to Pm dsRNA4-encoding protein, when using proteins extracted from ultracentrifugation fractionation as loading sample. Further PMF analysis of the same protein sample generated 9 peptide fragments, matching a partial sequence at aa 19–258 of the protein encoded by dsRNA6, accounting for 52% of the entire coverage (261aa) (Table S3). Other than these, no other proteins were found to be associated with the two mycoviruses identified in the present study. The obtained results suggested that the protein encoded by Pm dsRNA4 did exit and might function as a tentative structural component to unconventionally coat PdPmV1 dsRNA genome.

### Biological effects of PdPmV1 and PdNLV1 on *P. digitatum*

**Figure 3:**
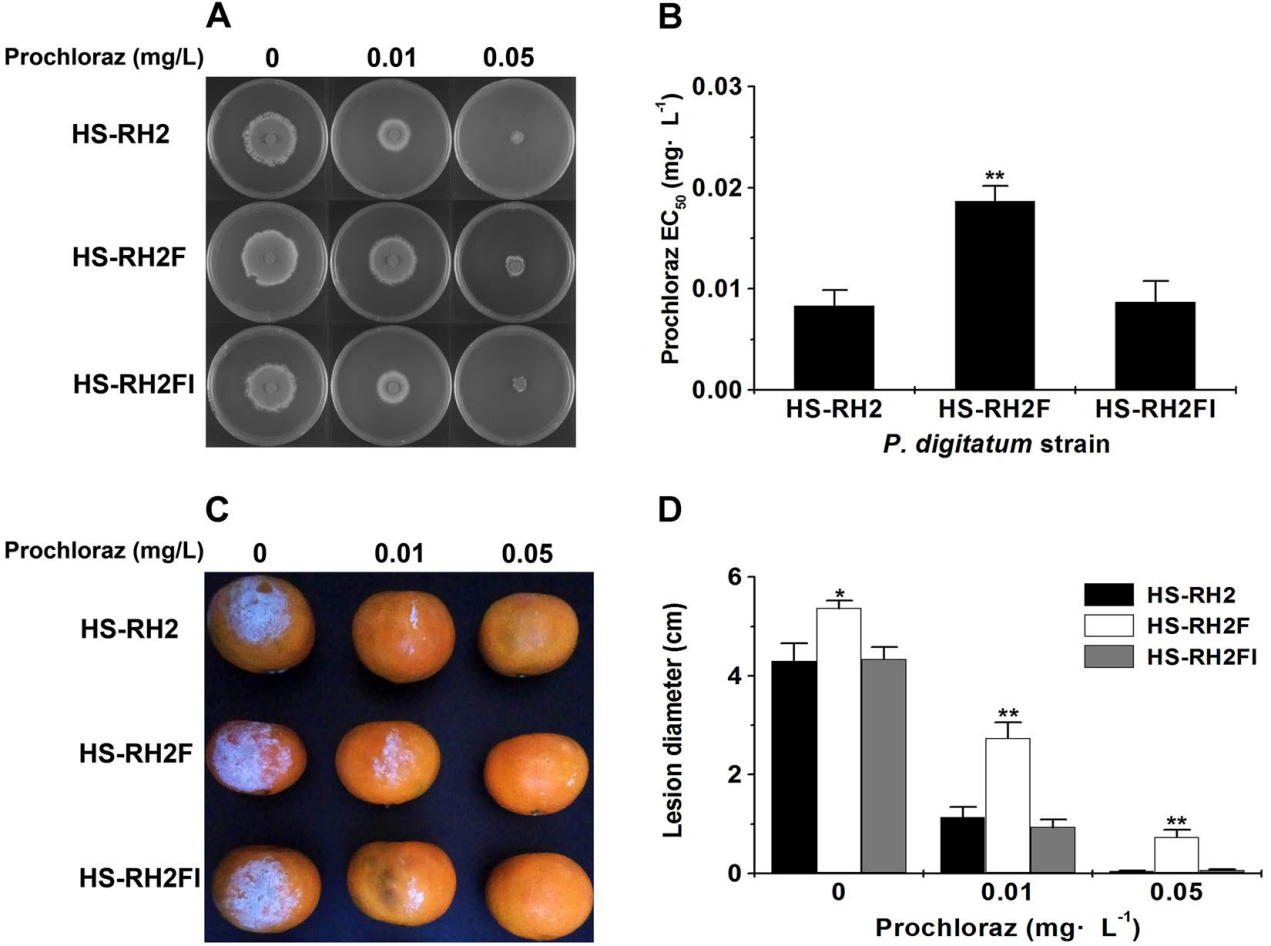
Assay of prochloraz resistance and related biological effects of *P. digitatum* strains derived from HS-RH2 including wild-type strain with PdPmV1 and PdNLV1 coinfection (HS-RH2), virus-cured strain (HS-RH2F) and virus-reinfected strain (HS-RH2FI). (**A**) Growth assay of *P. digitatum* HS-RH2, HS-RH2F and HS-RH2FI strains on PDA plates with or without prochloraz (concentrations: 0, 0.01 and 0.05 mg·L^−1^). All the strains were cultured at 25 °C for four days to calculate prochloraz EC_50_ values. (**B**) Comparison of prochloraz EC_50_ values of *P. digitatum* HS-RH2, HS-RH2F and HS-RH2FI strains. Each bar represents the EC_50_ value plus standard error of three measurements (**P < 0.01). (**C**) Virulence for *P. digitatum* HS-RH2, HS-RH2F and HS-RH2FI on the prochloraz-treated citrus peel (prochloraz concentrations: 0, 0.01 and 0.05 mg·L^−1^). The inoculated citrus fruits were incubated for a week at 25 °C. (**D**) Bars representing the mean lesion diameter of the virulence assay plus standard errors of three cultures are shown in panel C (*P < 0.05; **P < 0.01).

In order to assess the effects of mycoviruses on the fungal host, a virus-cured *P. digitatum* strain HS-RH2F was obtained from parental strain HS-RH2, using a combinational approach of protoplast regeneration and ribavirin treatment. However no colonies harboring just one of the viruses were recovered. The absence of PdPmV1 and PdNLV1 in HS-RH2F was confirmed by northern hybridization (Fig. 1B) and specific RT-PCR amplification (Fig. S4). The colony growth rate on PDA plates, EC_50_ value of prochloraz and virulence between the virus-free and virus-infected isolates were compared. As shown in Fig. 3A, comparing to HS-RH2F (virus-free), the parental strain HS-RH2 (virus-infected) exhibited little difference in colony growth rate at 0 mg·L^−1^ prochloraz condition but significantly lower growth rates in the presence of prochloraz (0.01 and 0.05 mg·L^−1^), indicating a virus-induced fungicide-conditioned hypovirulence. Such fungicide-conditioned hypovirulence was also reflected in the significantly lower EC_50_ value of prochloraz (Fig. 3B) and smaller lesion diameters on the prochloraz-treated citrus fruit epidermis (Fig. 3C,D) for HS-RH2 when comparing to those of HS-RH2F. When the HS-RH2F protoplasts were vertically transfected by sucrose- or CsCl-gradient-ultracentrifugation purified viral fraction (PdPmV1 and PdNLV1) or further purified naked dsRNA mixture (Pm dsRNA1-4 and NL1 dsRNA) from HS-RH2, the resulting HS-RH2FI showed highly similar phenotypes to HS-RH2 (Fig. 3). Such back-introduction experiments confirmed the role of virus-coinfection in the observed hypovirulence at fungicide treatments, i.e. the coinfection of PdPmV1 and PdNLV1 did lead to reduced prochloraz resistance for the host fungi.

**Figure 4:**
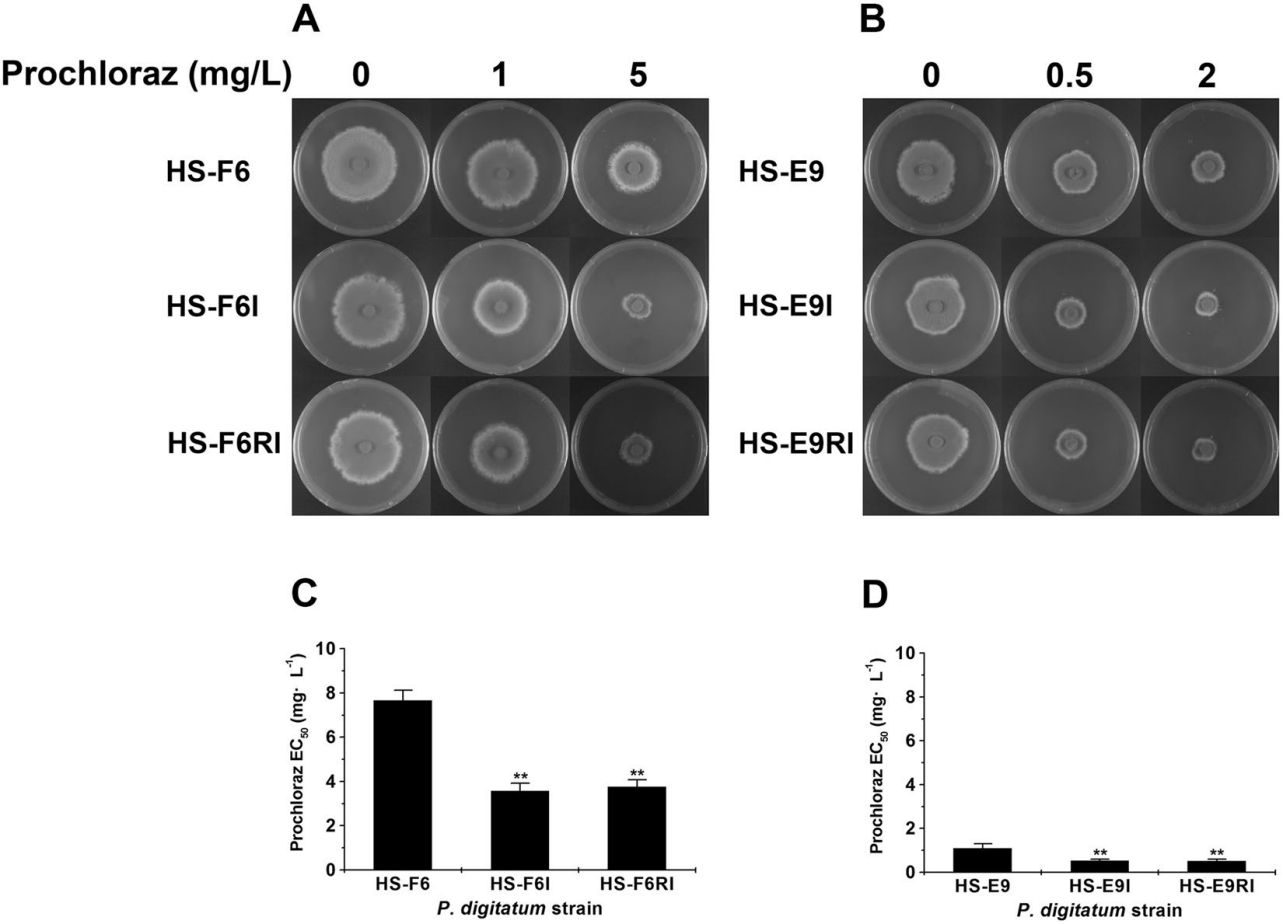
Comparison of prochloraz resistance of *P. digitatum* strains (HS-F6 and HS-E9) with or without coinfection by PdPmV1 and PdNLV1. (**A**) Growth assay of *P. digitatum* strains HS-F6 (virus-free), HS-F6I (coinfected by PdPmV1 and PdNLV1 from HS-RH2), and HS-F6RI (coinfected by PdPmV1 and PdNLV1 from HS-F6I) on PDA plates with or without prochloraz (concentrations: 0, 1 and 5 mg·L^−1^). (**B**) Growth assay of *P. digitatum* strains HS-E9 (virus-free), HS-E9I (coinfected by PdPmV1 and PdNLV1 from HS-RH2), and HS-E9RI (coinfected by PdPmV1 and PdNLV1 from HS-E9I) on PDA plates with or without prochloraz (concentrations: 0, 0.5 and 2 mg·L^−1^). All the strains were cultured at 25 °C for four days. (**C**) Comparison of prochloraz EC_50_ values of the *P. digitatum* strains HS-F6, HS-F6I and HS-F6RI. Each bar represents the EC_50_ value plus standard error of three measurements (**P < 0.01). (**D**) Comparison of prochloraz EC_50_ values of the *P. digitatum* strains HS-E9, HS-E9I and HS-E9RI. Each bar represents the EC_50_ value plus standard error of three measurements (**P < 0.01).

**Figure 5:**
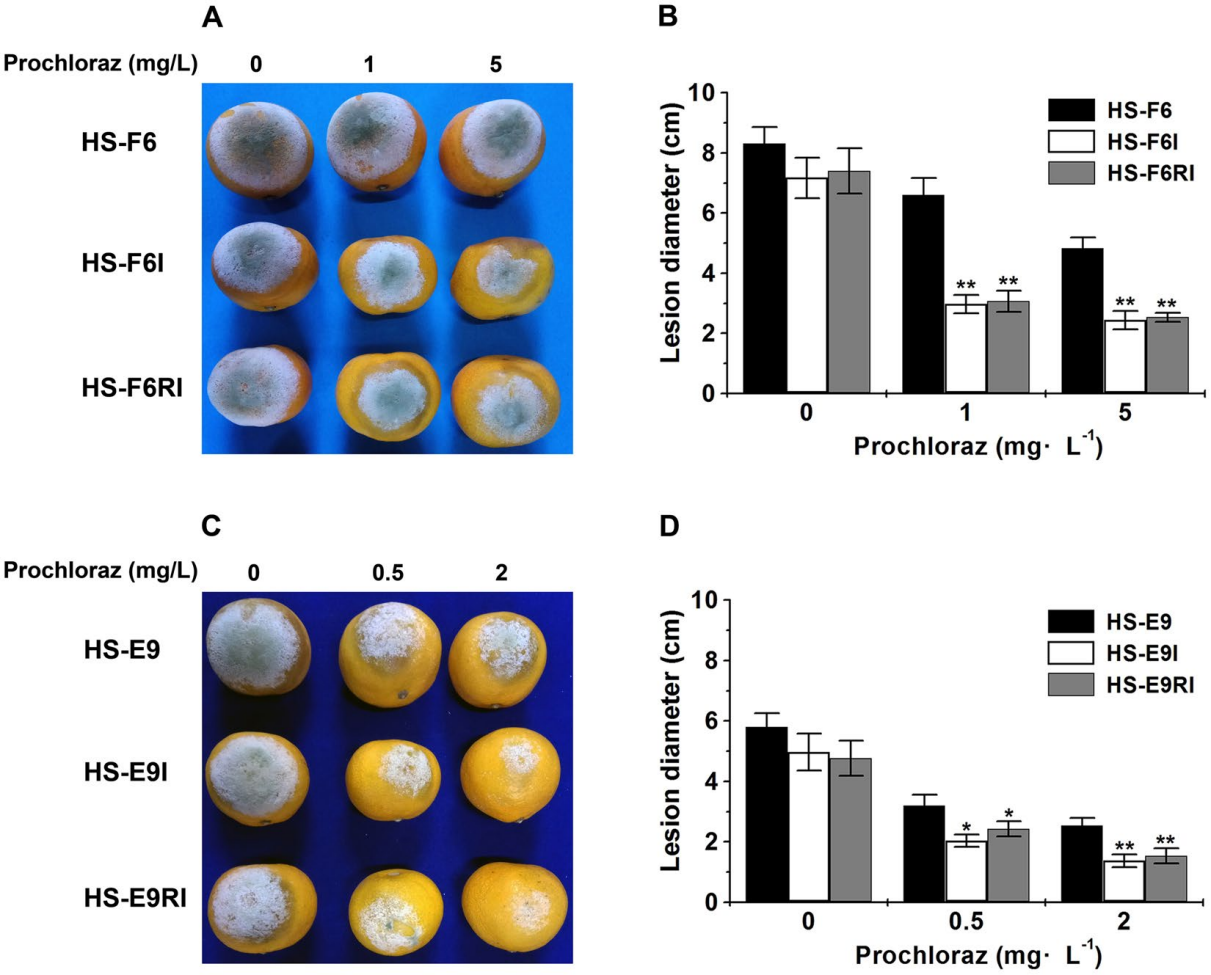
Biological effects of PdPmV1 and PdNLV1 coinfection on *P. digitatum* strains HS-F6 and HS-E9. (**A**) Virulence for HS-F6 and its virus-infected strains (HS-F6I and HS-F6RI) on the prochloraz-treated citrus peel (prochloraz concentrations: 0, 1 and 5 mg·L^−1^). The inoculated citrus fruits were incubated for 5 d at 25 °C. (**B**) Bars representing the mean lesion diameter of the virulence assay plus standard errors of three cultures are shown in panel A (**P < 0.01). (**C**) Virulence for HS-E9 and its virus-infected strains (HS-E9I and HS-E9RI) on the prochloraz-treated citrus peel (prochloraz concentrations: 0, 0.5 and 2 mg·L^−1^). The inoculated citrus fruits were incubated for 5 d at 25 °C. (**D**) Bars representing the mean lesion diameter of the virulence assay plus standard errors of three cultures are shown in panel C (*P < 0.05; **P < 0.01).

Here, it should be noted that all the *P. digitatum* strains investigated above, including HS-RH2, HS-RH2F and HS-RH2FI, were generally sensitive/susceptible to prochloraz according to EC_50_ range 0.01~0.02 mg·L^−1^. To further confirm the fungicide-conditioned hypovirulence, we selected two prochloraz-resistant and virus-free *P. digitatum* strains HS-F6 (high resistance with EC_50_~7.90 mg·L^−1^) and HS-E9 (relatively mild resistance with EC_50_~0.94 mg·L^−1^) as virus recipients to elucidate biological effects of PdPmV1 and PdNLV1 coinfection. These two virus-free strains were transfected by PdPmV1 and PdNLV1 (purified viral fraction after ultracentrifugation or naked dsRNA mixture from HS-RH2 as described in the preparation of HS-RH2FI) to obtain virus-infected strains HS-F6I and HS-E9I, respectively. In the absence of prochloraz, there was also no significant difference in colony morphology (Fig. 4A,B) and virulence (Fig. 5) between the virus-free and virus-infected strains. However, when treated with given concentration of prochloraz, the virus-infected strains (HS-F6I and HS-E9I) both markedly reduced their growth rates on PDA plates (Fig. 4A,B), indicating an obvious decrease in prochloraz resistance. Actually, the prochloraz EC_50_ value of HS-F6I and HS-E9I were significantly lower than that of HS-F6 and HS-E9, respectively (Fig. 4C,D). That is, 3.81 vs. 7.90 mg·L^−1^ for HS-F6I and HS-F6, and 0.64 vs. 0.94 mg·L^−1^ for HS-E9I and HS-E9. Moreover, the virulence of HS-F6I and HS-E9I directly on citrus fruit epidermis was also weaker than that of HS-F6 and HS-E9 in the presence of prochloraz (Fig. 5). Under 1 and 5 mg·L^−1^ prochloraz, the lesion diameter of HS-F6I was 2.70 cm and 1.53 cm, significant smaller than 3.07 cm and 2.73 cm of HS-F6, respectively (Fig. 5A,B). The similar experiment was performed to compare HS-E9I and HS-E9. The lesion diameter of HS-E9I was 2.93 cm and 2.23 cm under 0.5 and 2 mg·L^−1^ prochloraz, respectively; in contrast, the lesion diameter of HS-E9 was 3.50 cm and 3.17 cm at the same prochloraz concentration (Fig. 5C,D). Besides HS-F6 and HS-E9, more field isolates with different host backgrounds (i.e. high or mild prochloraz resistance), including HS-L17, HS-K25, HS-L8 and HS-K11, all exhibited the reduction of prochloraz resistance (i.e. the reduced EC_50_ values) when coinfected with PdPmV1 and PdNLV1 (Table S4). These results indicated a virus-induced reduction in prochloraz-resistance of the host *P. digitatum* strains.

In this work, using viral vertical transmission method, we further introduced PdPmV1 and PdNLV1 from donors HS-F6I and HS-E9I correspondingly back to their virus-free parental strains HS-F6 and HS-E9 (recipients). The resulting HS-F6RI and HS-E9RI, virus-infected isolates generated in the back-introduction virus transmission, exhibited highly similar phenotypes to their corresponding virus-donors, including colony morphology on PDA plates (Fig. 4A,B), prochloraz EC_50_ values (Fig. 4C,D), and virulence on citrus fruit epidermis (Fig. 5). The virus-back-introduction results with different host backgrounds further confirmed the virus-induced change of prochloraz resistance and the related fungicide-conditioned hypovirulence.

## Discussion

Mycoviruses are widely distributed in almost all fungal groups. However, statistical investigation on incidence of mycovirus infection towards specific fungal host(s) was not extensively documented. The earlier extensive searches of dsRNA mycoviruses were carried out for the host *C. parasitica* in North America, China and Japan^20–22^. Kondo *et al*. documented an extensive search for dsRNAs from field isolates of *R. necatrix*^23^. Zhong *et al*. reported 34 *U. virens* strains with dsRNA mycovirus infection in total 35 samples^24^. Recently, Kotta-Loizou and Coutts reported 16 *B. bassiana* isolates with such infection in total 75 samples from worldwide locations^10^. In this study, of all the 89 citrus postharvest pathogenic fungal strains from main citrus-growing areas in China, we have isolated 35 *P. digitatum* strains with mycovirus infection. The infection rates of 39.3% (35/89) suggested a well prevalence of dsRNA mycovirus infection in *P. digitatum* strains. According to partial sequencing analysis, 24 *P. digitatum* isolates are infected with mycoviruses, belonging to *Totiviridae* family and sharing great similarity with PdV1^17^, and 9 *P. digitatum* isolates are infected with mycoviruses, belonging to *Partitivirdae* family and sharing great similarity with PsV-S^24^. Here, mycoviruses coinfection was observed in two *P. digitatum* strains. One strain harbors two mycoviruses belonging to *Totiviridae* and *Partitivirdae* families which members have been well documented in many fungal strains^17,25^. The other strain (HB-36) harbors three mycoviruses, the one (PdV1) previously reported as *Totividae* member^17^ and the other two characterized as polymycovirus and Narna-like virus. Here, the member of Narna-like viruses and the member of polymycovirus were at first time molecularly identified in *Penicillium* strains.

An isolate HS-RH2 containing Pm and NL1 dsRNAs, i.e. dsRNA2-6 in order of electrophoresis mobility (Fig. S1E), was obtained through curing PdV1 dsRNA (dsRNA1 shown in Fig. S1E) from HB-36. The four dsRNA segments, labeled as Pm dsRNA1 to 4 (Fig. 1A), constitute the genome of PdPmV1, each containing a single ORF. The proteins encoded by PdPmV1 shared low aa sequence identities with those encoded by AfuPmV-1 and BdRV1. Notably, the unusually high GC contents were found in the full-length sequences of all that four dsRNAs with an average value of 59.9% and in their coding regions with an average value of 60.3%, which has been typically characterized in members of polymycoviruses^10^. Some conserved motifs, previously identified to locate in virus RNAs terminal regions with presumably important functions^12,25–27^, have also been observed in the 5′- and 3′-terminal sequences of PdPmV1 dsRNAs, exhibiting highly structured as predicted by mfold program. These two lines of evidences obtained from sequence analysis tentatively classified PdPmV1 as a polymycovirus.

Further, like all the reported polymycoviruses, the RdRp of PdPmV1, putatively encoded by Pm dsRNA1, contains a GDQN motif, a characteristic motif normally found in negative-strand ssRNA viruses of the order *Monoegavirales*^10,11,28^. Phylogenetic analysis has classified PdPmV1 as a polymycovirus, based on homolog alignment of selected RdRp sequences (Table S2). Actually, the members of newly proposed family ‘Polymycoviridae’ have close evolutionary proximity with positive-sense ssRNA viruses identified from *Caliciviridae* and *Astroviridae* families^10^. As a component of PdPmV1 genome, the Pm dsRNA2 putatively encodes a protein rich in arginine repeats (R-R, R-X-R, and R-R-R) that are functionally associated with endoplasmic reticulum (ER) retention signals, particularly involved in replication of positive-sense ssRNA viruses including caliciviruses and astroviruses^29,30^. These arginine-repeats-containing proteins have been implicated to favor dsRNA chaperones and/or replication machinery, achieving specific RNA virus life cycle^10,11^. In addition, a PAS-rich protein, initially described in the *Phlebiopsis gigantean* large virus 1^31^, was also deduced to be translated from the ORF of Pm dsRNA4. The proline-rich domain, frequently found in some scaffold proteins, has been functionally associated with protein-protein interactions, interacting with membrane components to assemble virus replication complexes^32,33^. The PAS-rich proteins encoded by corresponding segments of reported polymycoviruses are associated with the dsRNA genome of the virus and functioned in viral coating in an unconventional manner^11^.

In this study, the PASrp encoded by Pm dsRNA4 was identified by PMF analysis, and accordingly, no conventional virion was detected by TEM, although the whole genomic dsRNAs of PdPmV1 could be recovered from pellets obtained after sucrose gradient differentiation combined with ultracentrifugation. The results revealed that PdPmV1 is not conventionally escapsidated in virions, a similar phenomenon occurred for BbPmV-1^10^ and AfuPmV-1^11^. Zhai *et al*. reported “bacilliform virus-like particles” as presumable virions for polymycovirus BdRV1, but they did not correlate such viral particles with their designated cap proteins^12^. According to the methods described by Kotta-Loizou *et al*.^11^ and Zhai *et al*.^12^, we have also succeeded in purifying virus dsRNAs (Pm dsRNA1-4 and NL1 dsRNA) from HS-RH2. Such purified dsRNA mixture (naked PdPmV1 and PdNLV1 dsRNAs), verified by dsRNA electrophoresis profile, northern blotting, and further sequencing, could also be transfected as infectious entities to *P. digitatum* recipients including HS-RH2F, HS-F6 and HS-E9. However, we still failed to observe any viral or viral like particles under TEM scanning. Thus, all the polymycoviruses reported up to date including PdPmV1 (this study), BbPmV^10^, AfuTmV-1^11^ and BdRV1^12^ might exhibit a common feature that they have no or irregular encapsulating processes. For this reason, further studies are required to answer the questions how the protein conjugated with the viral dsRNAs and whether PdPmV1 presented in a form of unusual ribonucleoprotein.

At the same time, a novel mycovirus PdNLV1 whose genome corresponding to dsRNA5 was identified from *P. digitatum* strain HS-RH2. The dsRNA5-encoding protein showed 72% aa sequence identity with the RdRp encoded by BbSNLV, one case of dsRNA mycovirus in Narna-like viruses (or *Narnaviridae* family)^10^. Regarding dsRNA structure, multiple stem-loops at the 3′- and 5′-terminial sequences, exclusively identified in members of Narna-like viruses and narnaviruses^13^, were also detected in the secondary structure of PdNLV1 genomic dsRNA. In the present study, using RdRp sequences from members of *Narnaviridae* family and Narna-like viruses, we constructed one phylogenetic tree, placing PdNLV1 and BbSNLV into a clade evolutionally close with typical members of *Narnaviridae* family (Fig. 2F). The obtained results at least classified PdNLV1 as a Narna-like virus, and more research including subcellular localization analysis would be required to further clarify the evolution status of PdNLV1.

We have employed all available approaches to obtain an isolate solely infected with mycovirus PdPmV1 or PdNLV1, but failed. In the present study, using a specific fractionation of sucrose gradient ultracentrifugation or purified naked dsRNA mixtures, we only obtained the virus-coinfected fungal strains including HS-RH2FI, HS-F6I, HS-F6RI, HS-E9I and HS-E9RI, simultaneously containing Pm dsRNA1-4 and NL1 dsRNA (the genome and/or viral amplicons of PdPmV1 and PdNLV1 respectively). Meanwhile, through screening of virus-cured isolates, we only obtained fungal strains (HS-RH2F) simultaneously losing the mentioned mycoviruses (Pm dsRNA1-4 and NL1 dsRNA), i.e. also failing to obtain single cured progenies of HS-RH2. Our results that the naked viral dsRNAs possess infectious ability were in agreement with the previously reported polymycoviruses^10,11^. Nevertheless, the infection efficiency of the present naked dsRNA mixture was rather poor, i.e. about 2–3 targets per 100 subisolates regenerated from virus-transfected protoplasts. In addition, we would like to emphasize the fact that only all the naked PdPmV1 and PdNLV1 dsRNAs gathered as a whole did they have such infectious ability. The mechanism to explain these phenomena would be interesting and needs further research. Another issue to be highlighted was the introduction of PdNLV1 into *P. digitatum* isolates via the present virus-coinfection. To our best knowledge, this is the first report on the transfection of narnavirus or narna-like virus into typical filamentous fungi. The narnaviruses (ssRNA viruses) have been known as yeast nucleic acid elements and their fungal hosts been restrained to *Saccharomyces* yeasts^34–36^. However, unlike independent ability of narnavirus to infect yeasts, the transfection of PdNLV1 into *P. digitatum* strains, with no exception in this report, should cooperate with another virus PdPmV1, and more evidence are required to unravel the mechanism for such virus-coinfection.

In the absence of prochloraz, compared to HS-RH2, the virus-cured strain HS-RH2F exhibited little difference in growth rate on PDA plates (Fig. 3A) and lesion size at citrus fruit epidermis (Fig. 3C,D). In contrast, at 0.01 and 0.05 mg·L^−1^ prochloraz conditions, the virus-infected strains HS-RH2 and HS-RH2FI, both simultaneously containing PdPmV1 and PdNLV1, showed significantly lower fungicide EC_50_ values (Fig. 3B) and smaller lesion size at citrus fruit epidermis (Fig. 3C,D), indicating the virus-induced prochloraz-conditioned hypovirulent effects. In addition, the more virus-infected isolates HS-F6I, HS-F6RI, HS-E9I and HS-E9RI, all containing dsRNA elements from both PdPmV1 and PdNLV1, exclusively reduced their prochloraz-resistance on both PDA plates (Fig. 4) and citrus fruit epidermis (Fig. 5). The occurrence of prochloraz-conditioned hypovirulence in the multiple virus tranfectants with different host backgrounds verified the biological effects of PdPmV1 and PdNLV1 coinfection. Further, comparing the fungicide-relating phenotypes between parental strains (HS-F6I or HS-E9I) and virus back-introduced strains (HS-F6RI or HS-E9RI) in back-introduction experiments (Figs 4 and 5), also confirmed the virus-induced reduction of prochloraz resistance. In the past few years, more and more drug-resistant *P. digitatum* strains emerged, urgently requiring potential biological control agents. Previous reports supposed that DNA plasmids seems to modulate drug resistance of host fungi^37^, while no measurable effect of it has been observed up to our knowledge. The prochloraz-conditioned hypovirulent effect of mycoviruses, observed in this work, suggested the potential of these novel mycoviruses as enhancers for specific fungicide(s) to control citrus pathogenic fungi. According to our records on the prochloraz EC_50_ values of more virus-infected *P. digitatum* isolates (Table S4), interestingly, we found that the reduced fungicide resistance only occurred for the PdPmV1 and PdNLV1-coinfectants rather than any other virus infectant including the case of PdV1-infection^17^. This evidence associated the present prochloraz-conditioned hypovirulence with specific virus-host interaction(s). Nevertheless, as we have emphasized in the present study, it is hard to evaluate the contribution of single virus (independent PdPmV1 or PdNLV1) to the reduced prochloraz-resistance, and thus more field *P. digitatum* isolates including their virus-infected and virus-free progenies would be required to more definitely correlate prochloraz sensitivity to the mycoviruses.

In summary, the present work evaluated the prevalence of dsRNA elements in *Penicillium* isolates collected from main citrus-growing areas in China. In total, 39 of 152 *Penicillium* strains were infected by various mycoviruses. Among them, we at first time molecularly characterized PdPmV1 and PdNLV1 as polymycovirus and Narna-like virus, respectively. A decrease in prochloraz resistance was further reported in some representative *P. digitatum* strains with PdPmV1 and PdNLV1 coinfection, and the fungicide-conditioned hypovirulence related was also recorded.

## Materials and Methods

### Fungal strains and culture conditions

The *Penicillium* strains used in this study were isolated from decayed citrus fruit epidermis collected from markets, packing houses and citrus orchards in Hubei, Sichuan, Jiangxi and Yunnan province of China from 2015 to 2016. The rDNA ITS (internal transcript spacer) gene fragments of these strains were amplified and sequenced to confirm their identity. HS-RH2 was derived from HB-36 through curing the virus consisting of dsRNA1. HS-F6I and HS-E9I were derived from HS-F6 and HS-E9 respectively by transfection of PdPmV1 and PdNLV1. HS-RH2F was a virus-cured strain derived from HS-RH2, and the absence of virus dsRNA elements was assessed by agarose gel electrophoresis and northern blot. All the strains were cultured on potato dextrose agar (PDA) medium or in potato dextrose broth (PDB, PDA without agar) on a rotary shaker (180 rpm) at 25 °C.

### dsRNA extraction and purification

The mycelia were harvested and homogenized in liquid nitrogen, and dsRNA was isolated by CF-11 cellulose (Sigma, St. Louis, MO, USA) column chromatography following published procedure^38^. The dsRNA preparation was further digested with S1 nuclease (TaKaRa, Dalian, China) and subsequently with RNase-free DNase I (TaKaRa, Dalian, China), electrophoresed on a 1% agarose gel, and then visualized by staining with ethidium bromide. The dsRNA was excised and purified with a gel extraction kit (Axygen, Suzhou, China), dissolved in RNase-free water, and kept at −70 °C until use.

### Nucleotide sequencing

A total of 5 μg purified dsRNA was subjected to library preparation following the a reported protocol^39^ and then used for high-throughput deep-sequencing using the Illumina platform. Sequenced reads match with the host *P. digitatum* databases (isolate Pd1) were expurgated and the remaining reads were de novo assembled using Velvet (version 1.2.10) with default parameters^40^. All contigs were subjected to BLASTN and BLASTX alignments against the NCBI database to search for viral sequences. The 5′- and 3′-terminal sequences were further determined using RACE-PCR. Firstly, the 3′-terminus of each strand of dsRNA was ligated to the closed adaptor primer PC3-T7 loop with a phosphorylated (p) 5′ end and an NH_2_ 3′ end (5′-p-GGATCCCGGGAATTCGGTAATACGACTCACTATATTTTTATAGTGAGTCGTATT A-OH-3′) by T4 RNA ligase (TaKaRa, Dalian, China) at 16 °C for 18 h. Then, the oligonucleotide-ligated dsRNA was reverse transcribed by a primer complementary to the oligonucleotide used for the RNA ligation (PC2 [5′-CCGAATTCCCGGGATCC-3′]) and sequence-specific primers corresponding to the 5′- and 3′-terminal sequences of the dsRNA in presence of M-MLV reverse transcriptase according to previously described protocol^41^. The amplified cDNA products were sequenced and subsequently assembled. The full-length sequences of the dsRNAs were further confirmed by RT-PCR with specific primers and sequencing. Northern blot hybridization analysis was performed as previously described^42^. The specific probes (~500 bp) were labeled with digoxigenin and used to probe the RNA blot. The positions of the probes were indicated in Figs 1C and 2C.

### Bioinformatic and phylogenetic analysis

The obtained nucleotide sequences were subjected to BLAST program on the NCBI website to see the sequence similarity. Potential ORFs were deduced and translated using the DNAMAN software package with the default parameters and the ORF finder on the NCBI website^43^. Multiple sequence alignments of nucleotide and amino acid sequences were generated using the CLUSTAL_X program^44^. The phylogenetic trees for RdRps were constructed using the neighbor-joining method of MEGA version 6 software with bootstrapping analysis of 1000 replicates^45^.

### Virus particle purification and peptide mass fingerprinting (PMF) analysis

To isolate the virus-like particles (VLPs), approximately 100 g mycelia (wet weight) were harvested and mixed with 400 mL of 50 mmol·L^−1^ sodium phosphate buffer (pH 7.4), and were then ground into homogenate. The homogenate was subjected to differential centrifugation and ultracentrifugation in sucrose density gradients (200 to 600 mg·mL^−1^ with intervals of 100 mg·mL^−1^) and CsCl density gradients, respectively as described by the previously reported methods^11,12^. Aliquots about 1 mL were individually collected and subjected to UV absorbance assessment, and the fractions with a peak absorption were subjected to ultracentrifugation. The obtained VLPs were suspended in 100 μL of 0.01 M sodium phosphate buffer (pH 7.4) and then detected by SDS-PAGE analysis, transmission electron microscopy (TEM) and used for infectivity assays. The gel stripe with protein band on 12% (wt/vol) polyacrylamide gel was excised and subjected to peptide mass fingerprinting (PMF) analysis at ProtTech, Inc. (Suzhou, China). All the processes described above were performed as previously described with minor modifications^46,47^.

### Preparation of virus-cured *P. digitatum* strains

To eliminate the mycoviruses from the host fungus, we treated the target strains with ribavirin as previously described^48–50^. Based on the ribavirin protocol, we exploited HS-B36 as original strain to generate a curing library containing a large quantity of virus-cured and virus-partial-cured isolates, and finally got desirable isogenic strains HS-RH2 just lacking dsRNA1 element by library screening (not more than 10 target isolates per one round screening). Five biological replicates of HS-RH2 were set for further research. To cure mycoviruses PdPmV1 and PdNLV1 from their host HS-RH2, the conidia of HS-RH2 were incubated in PDB media containing 100 mmol·L^−1^ ribavirin for 10 h to generate virus-free progenies (HS-RH2F). For each parental strain HS-RH2, five biological replicates of HS-RH2F were selected for further experiments.

### Protoplast transfection

The virus-free *P. digitatum* strains HS-F6 and HS-E9 were used as the recipients in the transfection experiments. Protoplasts of HS-F6 and HS-E9 strains, each containing five biological replicates, were prepared and mixed with the purified viral particles by Ultrafree-MC sterile centrifugal filters (Pall Corporation, America). In the PTC buffer (40% PEG 3350, 100 mmol·L^−1^ Tris-HCl, 100 mmol·L^−1^ CaCl_2_, pH8.0), the purified virus particles and further purified naked dsRNA mixture containing PdPmV1 and PdNLV1 genome RNA elements were respectively introduced into the protoplasts of HS-F6 and HS-E9 in the presence of polyethylene glycol (PEG) according to the protocol as previously reported^11,48,51,52^. Transfected protoplasts were coated onto regeneration media (0.7 mol·L^−1^ sucrose, 0.5 g·L^−1^ yeast extract, 15 g·L^−1^ agar) plates and incubated until single colony regeneration at 25 °C. The desirable transfectants HS-F6I and HS-E9I were selected and verified by dsRNA extraction and RT-PCR amplification with specific primer pairs. For each virus-infected strain (HS-F6I or HS-E9I), five biological replicates were prepared for further biological tests.

### Assays of vegetative growth, prochloraz EC_50_ and virulence

The conidial suspension (50 μL, 1 × 10^6^ spores mL^−1^) of the *P. digitatum* strains was coated onto a PDA plate and incubated at 25 °C for 24 h. Mycelia plugs (approximately 0.8 cm in diameter) were obtained from the plate using a punch. The mycelia plugs were placed on new PDA plates and cultivated for 4 d at 25 °C or according to the experimental need, and the diameters of different colonies were measured. EC_50_ values of prochloraz for *P. digitatum* strains were measured according to previous reports^6,47^. The diameters of the colonies cultivated on PDA with different concentrations of prochloraz were measured and the average of the colony diameters in each independent test was used for EC_50_ calculation using SPSS software (version 10.0). Virulence assays were performed directly on citrus fruits. The citrus fruits were washed with distilled water to remove the contamination. A 2 mm hole was made on the pericarp using a 1 mL pipette tip, and a 3 μL conidial suspension was injected (10^6^ spores mL^−1^). The diameters of the disease spots were measured and compared after incubation for 5d at 25 °C or according to the experimental need.

## Electronic supplementary material

Supplimentary Materials
