# Supplementary material for: Characterization of two novel mycoviruses from Penicillium digitatum and the related fungicide resistance analysis
Source: Sci Rep. 2018 Apr 3;8:5513. doi: 10.1038/s41598-018-23807-3 (PMC5882929; doi:10.1038/s41598-018-23807-3)
Supplement: Supplementary file 1 — Supplimentary Materials [file 41598_2018_23807_MOESM1_ESM.docx]

**Supplimentary Materials**

**Characterization of two novel mycoviruses from *Penicillium digitatum* and the related fungicide resistance analysis**

Yuhui Niu**^+^**, Yongze Yuan**^+^**, Jiali Mao, Zhu Yang, Qianwen Cao, Tingfu Zhang, Shengqiang Wang, Deli Liu*


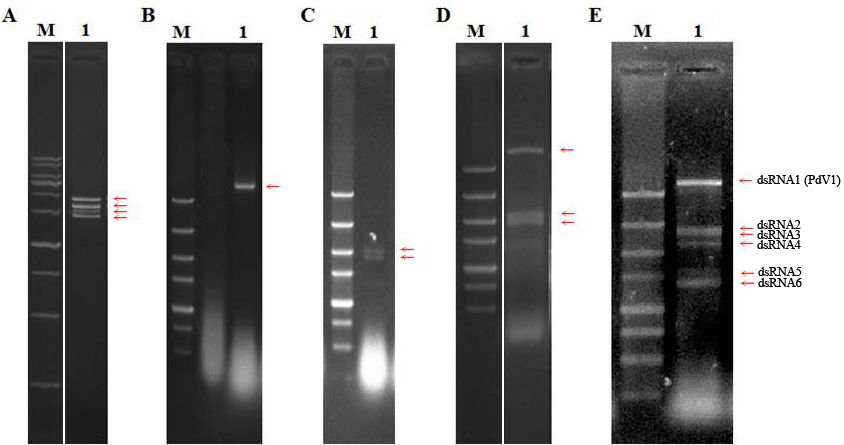


**Figure S1 Representative dsRNA patterns detected from *Penicillium* isolates. (A)** Agarose gel electrophoresis of dsRNAs extracted from YN-1 containing 4 dsRNA segments (Lane 1). Lane M, DNA size marker (1kb Marker, TaKaRa, Dalian, China). **(B)** Agarose gel electrophoresis of dsRNAs extracted from HB-1 containing 1 dsRNA segment (Lane 1). Lane M, DNA size marker (DS 5000, TaKaRa, Dalian, China). **(C)** Agarose gel electrophoresis of dsRNAs extracted from HB-11 containing 2 dsRNA segments (Lane 1). Lane M, DNA size marker (DS 5000, TaKaRa, Dalian, China). **(D)** Agarose gel electrophoresis of dsRNAs extracted from HB-22 containing 3 dsRNA segments (Lane 1). Lane M, DNA size marker (DS 5000, TaKaRa, Dalian, China). **(E)** Agarose gel electrophoresis of dsRNAs extracted from HB-36 containing 6 dsRNA segments (Lane 1). Lane M, DNA size marker (DS 5000, TaKaRa, Dalian, China). All the dsRNA segments were indicated by red arrow.


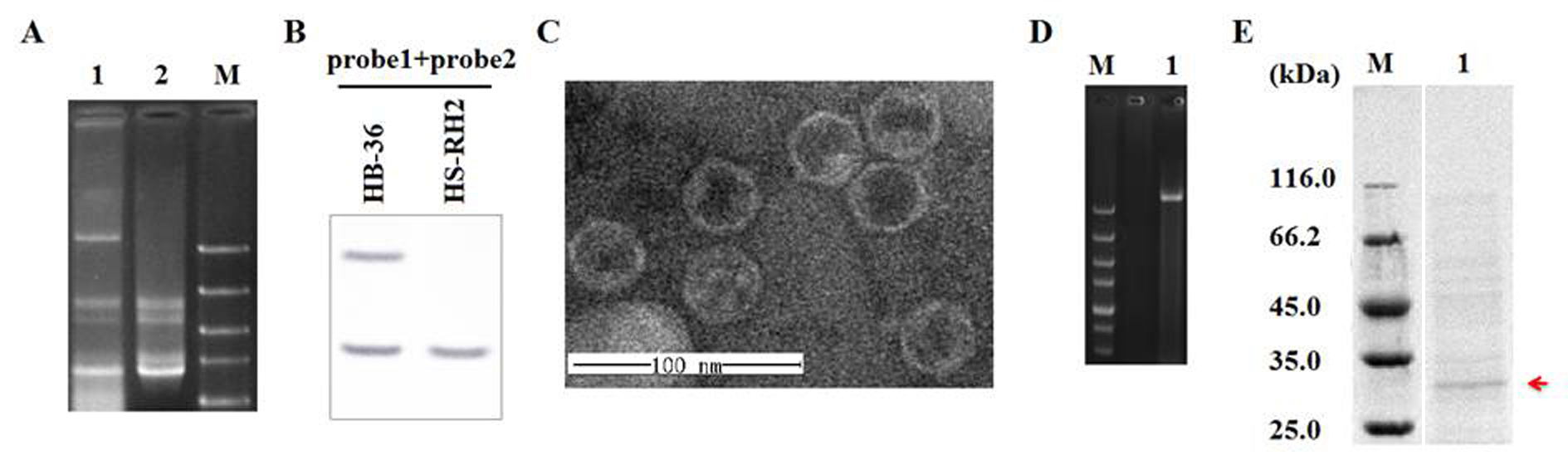


**Figure S2 Virus detection in *P. digitatum* HB-36 and HS-RH2. (A)** Electrophoretic profile on a 1% agarose gel of dsRNA preparations extracted from *P. digitatum* isolates HB-36 (Lane 1) and HS-RH2 (Lane 2). Lane M, DNA size marker (DS 5000, TaKaRa, Dalian, China). **(B)** Northern blot hybridization of dsRNA extracted from *P. digitatum* isolates HB-36 and HS-RH2 using probe1 and probe2 respectively specific for dsRNA1 (PdV1 dsRNA) and dsRNA2 (Pm dsRNA1 in Fig. 1A) (Figure S8). **(C)** Electron micrograph of virus particles isolated from HB-36. **(D)** Nucleic acids were extracted from virus particles by the SDS-phenol method, electrophoresed in a 1% agarose gel, and stained with ethidium bromide (lane 1). Lane M, DNA size marker (DS 5000, TaKaRa, Dalian, China). **(E)** Protein components of purified virus preparations of HS-RH2 (Lane1). Lane M, PageRuler™ Unstained Protein Ladder (Thermo Fisher Scientiﬁc, USA).


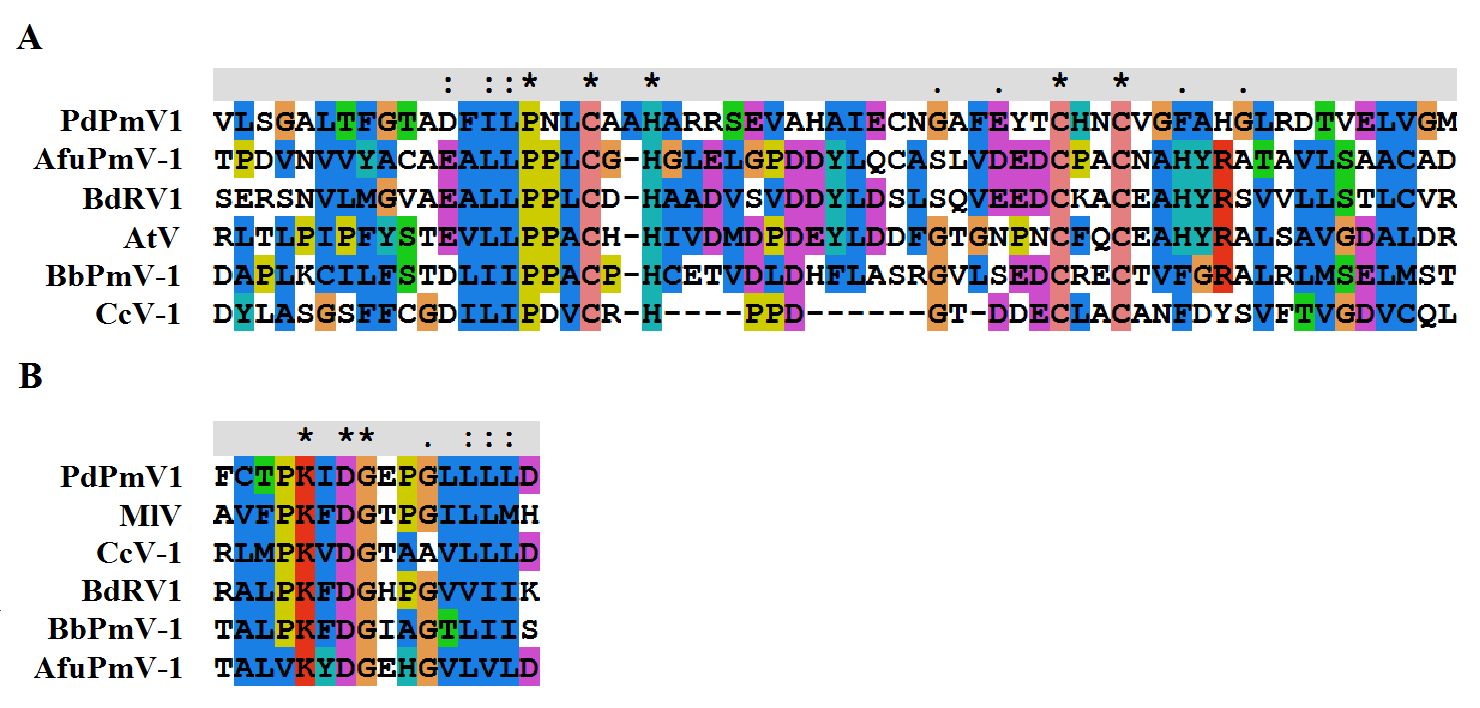


**Figure S3 (A)** Amino acid sequence alignment of cysteine-rich zinc finger-like motifs found in the protein of unknown function putatively encoded by Pm dsRNA2, and proteins encoded by the other reported dsRNA2 of polymycoviruses listed in Table S5. **(B)** Amino acid sequence alignment of the catalytic methyltransferase motifs detected in the protein putatively encoded by Pm dsRNA3, and proteins encoded by the other reported dsRNA3 of polymycoviruses listed in Table S5. Asterisks signified identical amino acid residues, and colons signified highly conserved residues and single dots signified less conserved but related residues.


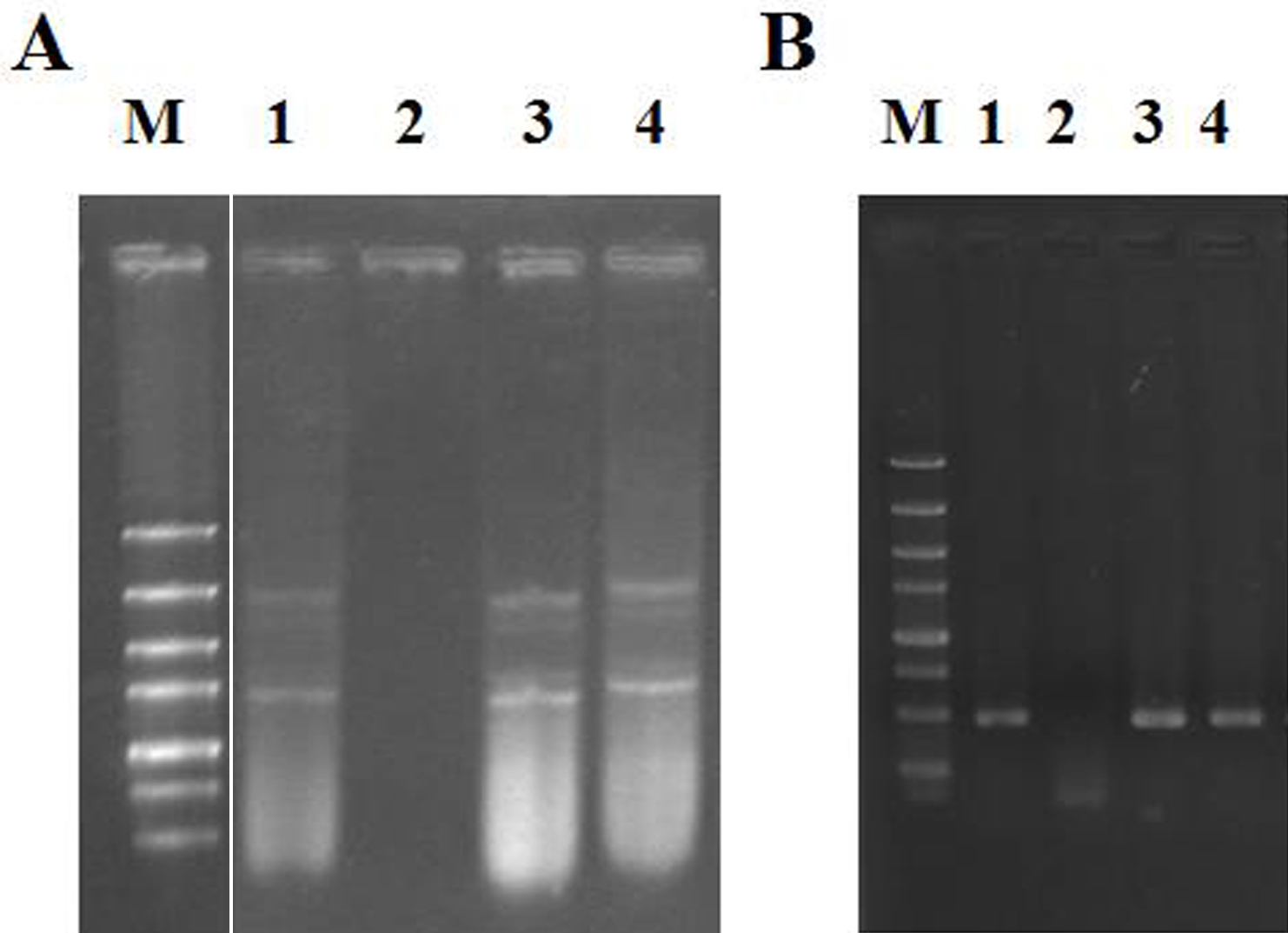


**Figure S4 dsRNAs detection in HS-RH2, HS-RH2F, HS-F6I and HS-E9I. (A)** PdPmV1 dsRNAs extracted from wild-type (HS-RH2) (Lane1), virus-free (HS-RH2F) (Lane2) and transfected cultures (HS-F6I and HS-E9I) isolates (Lane3 and Lane4). **(B)** RT-PCR amplification of a 337-bp segment from Pm dsRNA1 from equal amounts of dsRNA extracted from wild-type (HS-RH2) (Lane1), virus-free (HS-RH2F) (Lane2) and transfected cultures (HS-F6I and HS-E9I) isolates (Lane3 and Lane4). Lane M, DNA size marker (DS 5000, TaKaRa, Dalian, China).


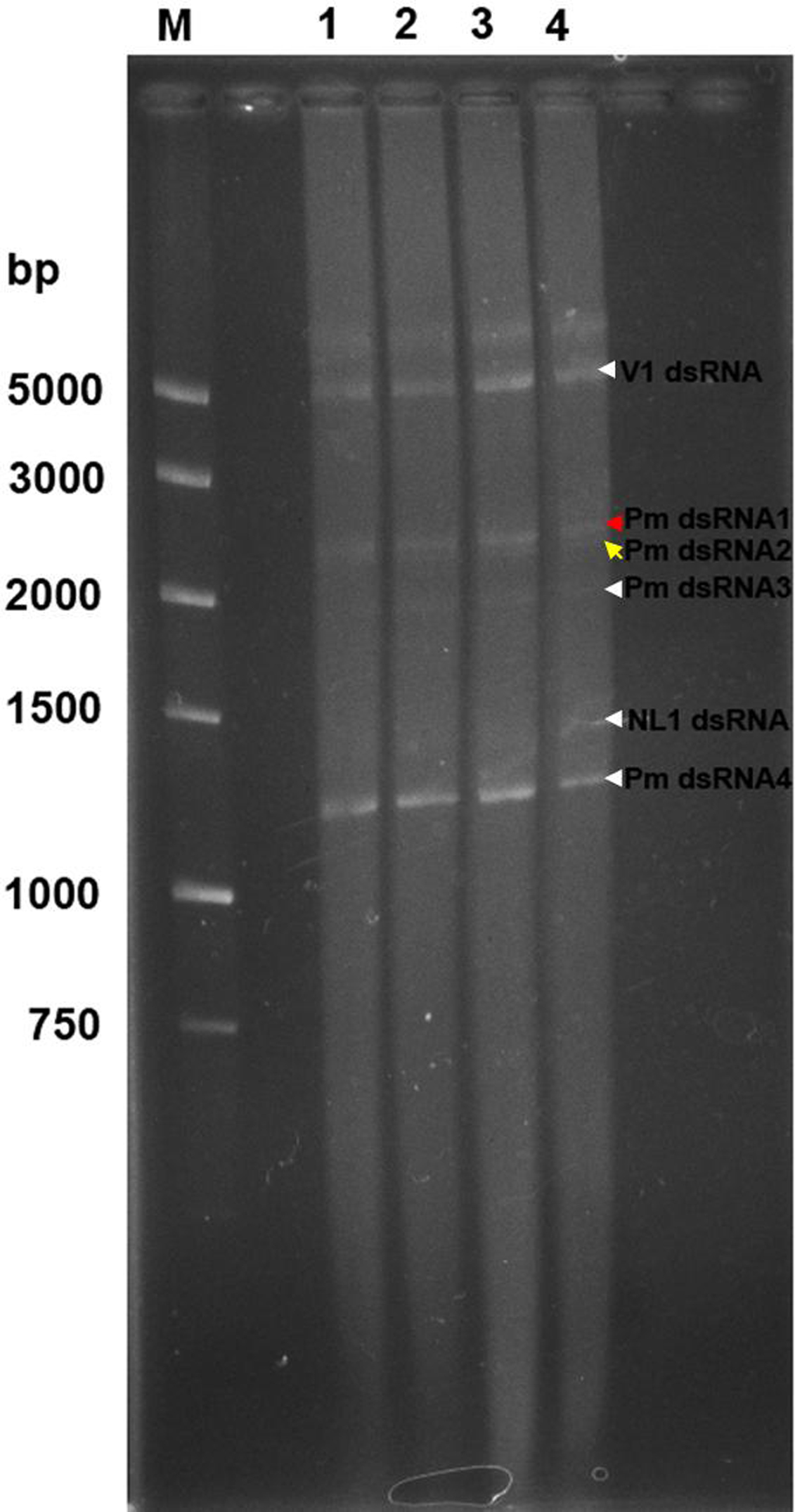


**Figure S5** The electrophoresis using 2% (w/v) agarose gel to isolate dsRNA preparations from *P. digitatum* isolate HB-36. Lanes 1-4 represented 4 replicates for HB-36 dsRNA extracts and lane M represented DNA size marker (DS 5000, TaKaRa, Dalian, China). The Pm dsRNA1 and 2, only partially separated by 2% (w/v) agarose gel, were indicated by red and yellow arrow, respectively, and other dsRNAs (i.e. V1 dsRNA, NL1 dsRNA, Pm dsRNA3 and 4) were indicated by white arrows.


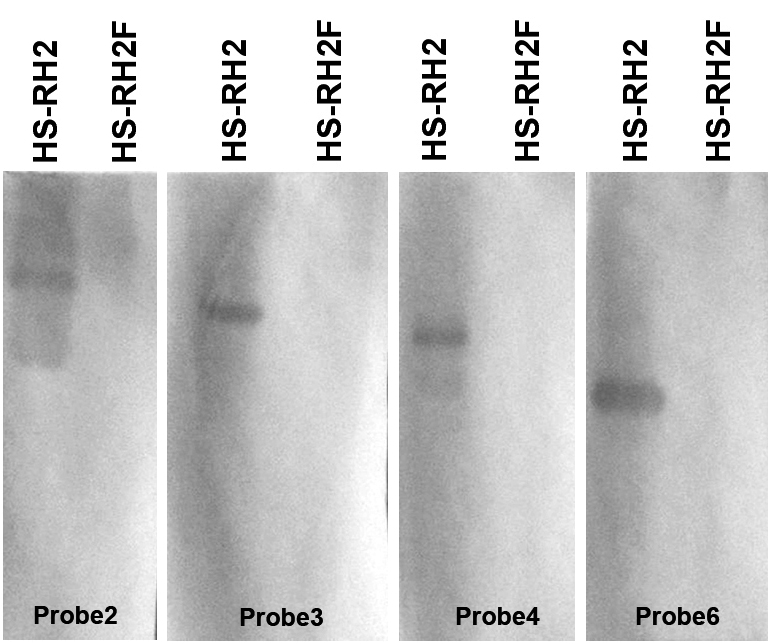


**Figure S6** The full-length and digital-compliable images for the northern blots of PdPmV1 dsRNAs in HS-RH2 and HS-RH2F, corresponding to **Figure 1 panel B**. The dsRNAs were separated under denaturing electrophoresis conditions, blotted onto nylon membranes, and probed by DIG-labeled DNA fragments (i.e. probes 2, 3, 4 and 6 respectively applied to blot Pm dsRNA1 to 4).


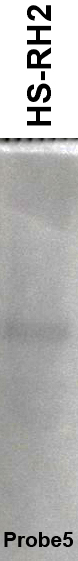


**Figure S7** The full-length and digital-compliable image for the northern blot of PdNLV1 dsRNA in HS-RH2, corresponding to **Figure 2 panel B**. The dsRNA generated from PdNLV1 was separated under denaturing electrophoresis conditions, blotted onto nylon membranes, and probed by DIG-labeled DNA fragment (probe5).


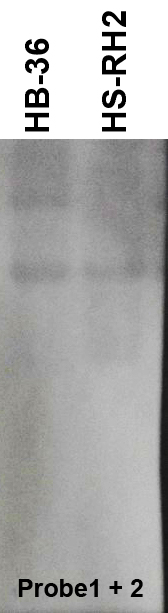


**Figure S8** The full-length and digital-compliable image corresponding to **Figure S2 panel B**. The northern blots of dsRNA(s) from *P. digitatum* isolates HB-36 and HS-RH2 with V1 dsRNA- and Pm dsRNA1-specific probes (i.e. probe1 and probe2, respectively).

**Table S1** Information on *Penicillium* isolates containing dsRNA elements.

| Isolate name | Species | Location | dsRNA elements (kbp) | Mycovirus family |
| --- | --- | --- | --- | --- |
| HB-1 | *P. digitatum* | Hubei | 5.2 | *Totiviridae* |
| HB-2 | *P. digitatum* | Hubei | 5.2 | *Totiviridae* |
| HB-4 | *P. digitatum* | Hubei | 5.2 | *Totiviridae* |
| HB-5 | *P. digitatum* | Hubei | 5.2 | *Totiviridae* |
| HB-6 | *P. digitatum* | Hubei | 5.2 | *Totiviridae* |
| HB-7 | *P. digitatum* | Hubei | 5.2 | *Totiviridae* |
| HB-9 | *P. digitatum* | Hubei | 5.2 | *Totiviridae* |
| HB-11 | *P. digitatum* | Hubei | 2.0, 1.8 | *Partitiviridae* |
| HB-12 | *P. digitatum* | Hubei | 2.0, 1.8 | *Partitiviridae* |
| HB-19 | *P. digitatum* | Hubei | 2.0, 1.8 | *Partitiviridae* |
| HB-21 | *P. digitatum* | Hubei | 2.0, 1.8 | *Partitiviridae* |
| HB-22 | *P. digitatum* | Hubei | 5.2, 2.0, 1.8 | *Totiviridae*, *Partitiviridae* |
| HB-23 | *P. digitatum* | Hubei | 2.0, 1.8 | *Partitiviridae* |
| HB-28 | *P. digitatum* | Hubei | 2.0, 1.8 | *Partitiviridae* |
| HB-29 | *P. digitatum* | Hubei | 5.2 | *Totiviridae* |
| HB-30 | *P. digitatum* | Hubei | 5.2 | *Totiviridae* |
| HB-36 | *P. digitatum* | Hubei | 5.2 | *Totiviridae* |
|  |  |  | 2.3, 2.3, 2.0, 1.3 | ‘Polymycoviridae’ |
|  |  |  | 1.6 | Narna-like viruses |
| HB-37 | *P. digitatum* | Hubei | 5.2 | *Totiviridae* |
| HB-38 | *P. digitatum* | Hubei | 5.2 | *Totiviridae* |
| SC-3 | *P. digitatum* | Sichuan | 5.2 | *Totiviridae* |
| SC-9 | *P. digitatum* | Sichuan | 5.2 | *Totiviridae* |
| SC-11 | *P. digitatum* | Sichuan | 2.0, 1.8 | *Partitiviridae* |
| SC-12 | *P. crustosum* | Sichuan | 3.6, 3.2, 3.0, 2.9 | *Chrysoviridae* |
| SC-19 | *P. crustosum* | Sichuan | 3.6, 3.2, 3.0, 2.9 | *Chrysoviridae* |
| SC-20 | *P. crustosum* | Sichuan | 3.6, 3.2, 3.0, 2.9 | *Chrysoviridae* |
| SC-32 | *P. digitatum* | Sichuan | 2.0, 1.8 | *Partitiviridae* |
| SC-33 | *P. digitatum* | Sichuan | 5.2 | *Totiviridae* |
| SC-38 | *P. digitatum* | Sichuan | 5.2 | *Totiviridae* |
| SC-39 | *P. digitatum* | Sichuan | 5.2 | *Totiviridae* |
| SC-50 | *P. digitatum* | Sichuan | 2.0, 1.8 | *Partitiviridae* |
| SC-59 | *P. digitatum* | Sichuan | 5.2 | *Totiviridae* |
| SC-60 | *P. digitatum* | Sichuan | 5.2 | *Totiviridae* |
| SC-63 | *P. digitatum* | Sichuan | 5.2 | *Totiviridae* |
| SC-66 | *P. digitatum* | Sichuan | 5.2 | *Totiviridae* |
| SC-74 | *P. digitatum* | Sichuan | 5.2 | *Totiviridae* |
| SC-77 | *P. digitatum* | Sichuan | 5.2 | *Totiviridae* |
| SC-100 | *P. digitatum* | Sichuan | 5.2 | *Totiviridae* |
| SC-103 | *P. digitatum* | Sichuan | 5.2 | *Totiviridae* |
| YN-1 | *P. italicum* | Yunnan | 3.6, 3.2, 3.0, 2.9 | *Chrysoviridae* |

**Table S2** Information on the virus isolates used for sequence alignment and phylogenetic analysis of their RdRps.

| Virus name | Abbreviation | GenBank  accession no. | Family | Genus |
| --- | --- | --- | --- | --- |
| Duck *astrovirus* C-NGB | DaV C-NGB | YP_002728002 | *Astroviridae* | *Avastrovirus* |
| *Avastrovirus* 1 | AV1 | CAB95006 | *Astroviridae* | *Avastrovirus* |
| Avian nephritis virus 1 | AnV1 | BAA92848 | *Astroviridae* | *Avastrovirus* |
| Goose *astrovirus* | GaV | KY271027 | *Astroviridae* | *Avastrovirus* |
| *Avastrovirus* 3 | AV3 | AAF60952 | *Astroviridae* | *Avastrovirus* |
| *Mamastrovirus* 10 | MV10 | AAO32082 | *Astroviridae* | *Mamastrovirus* |
| *Mamastrovirus* 13 | MV13 | CAB95003 | *Astroviridae* | *Mamastrovirus* |
| Mink *astrovirus* | MaV | AY179509 | *Astroviridae* | *Mamastrovirus* |
| Feline *astrovirus* 2 | FaV2 | AGT99974 | *Astroviridae* | *Mamastrovirus* |
| Qinghai Himalayan marmot *astrovirus* 2 | QhhmaV2 | KY024238 | *Astroviridae* | *Mamastrovirus* |
| Qinghai Himalayan marmot *astrovirus* 1 | QhhmaV1 | KY024237 | *Astroviridae* | *Mamastrovirus* |
| Norwalk virus | NoV | M87661 | *Caliciviridae* | *Norovirus* |
| Murine *norovirus* 1 | MnV | DQ285629 | *Caliciviridae* | *Norovirus* |
| *Norovirus* GIII | NV GIII | JX145650 | *Caliciviridae* | *Norovirus* |
| Norwalk-like virus | NLV | AB044366 | *Caliciviridae* | *Norovirus* |
| *Norovirus* Hu | NV Hu | JQ613567 | *Caliciviridae* | *Norovirus* |
| Canine *calicivirus* | CcV | AB070225 | *Caliciviridae* | *Vesivirus* |
| Walrus *calicivirus* | WcV | AF321298 | *Caliciviridae* | *Vesivirus* |
| Vesicular exanthema of swine virus | VesV | NP_786896 | *Caliciviridae* | *Vesivirus* |
| Feline *calicivirus* | FcV | L40021 | *Caliciviridae* | *Vesivirus* |
| Rabbit *vesivirus* | RvV | AJ866991 | *Caliciviridae* | *Vesivirus* |
| *Sapovirus* Mc10 | SV Mc10 | AY237420 | *Caliciviridae* | *Sapovirus* |
| Porcine enteric *sapovirus* | PesV | NP_051035 | *Caliciviridae* | *Sapovirus* |
| *Sapovirus* Hu | SV Hu | AY694184 | *Caliciviridae* | *Sapovirus* |
| Sapovirus 12 | SV12 | AY603425 | *Caliciviridae* | *Sapovirus* |
| Bat *sapovirus* TLC58/HK | BsV TLC58 | JN899075 | *Caliciviridae* | *Sapovirus* |
| [European brown hare syndrome virus](https://www.ncbi.nlm.nih.gov/Taxonomy/Browser/wwwtax.cgi?id=33756) | EBHSV | Z69620 | *Caliciviridae* | *Lagovirus* |
| Rabbit hemorrhagic disease virus | RhV | NP_062875 | *Caliciviridae* | *Lagovirus* |
| Rabbit hemorrhagic disease virus-FRG | RhV FRG | M67473 | *Caliciviridae* | *Lagovirus* |
| Rabbit *calicivirus* Australia 1 MIC-07 | RcaV MIC-07 | EU871528 | *Caliciviridae* | *Lagovirus* |
| Newbury agent 1 | NaV 1 | DQ013304 | *Caliciviridae* | *Nebovirus* |
| bovine *calicivirus* NB | BcV NB | NP_663315 | *Caliciviridae* | *Nebovirus* |
| Bovine *calicivirus* Kir. | BcV Kir. | KT119483 | *Caliciviridae* | *Nebovirus* |
| *Calicivirus* isolate TCG | CV TGG | AB117797 | *Caliciviridae* | *Nebovirus* |
| *Cladosporium* *cladosporioides* virus1 | CcV1 | AII80567 | ‘Polymycoviridae’ | unassigned |
| *Aspergillus fumigatus* tetramycovirus-1 | AfPmV-1 | CDP74618 | ‘Polymycoviridae’ | unassigned |
| *Alternaria tenuissima* virus | AtV | AJP08049 | ‘Polymycoviridae’ | unassigned |
| *Beauveria bassiana* polymycovirus-1 | BbPmV1 | LN896307 | ‘Polymycoviridae’ | unassigned |
| *Botryosphaeria dothidea* RNA virus 1 | BdRV1 | YP_009342446 | ‘Polymycoviridae’ | unassigned |
| *Beauveria bassiana* small Narna-like virus | BbSNLV | LT627647 | *Narnaviridae* | *Narnavirus* |
| *Leptomonas seymouri* Narna-like virus 1 | LsNLV1 | ANI86034 | *Narnaviridae* | *Narnavirus* |
| *Saccharomyces cerevisiae* 20S RNA *narnavirus* | ScNV 20S | AAC98925 | *Narnaviridae* | *Narnavirus* |
| *Saccharomyces cerevisiae* 23S RNA *narnavirus* | ScNV 23S | AAC98708 | *Narnaviridae* | *Narnavirus* |
| *Fusarium poae narnavirus* 1 | FpNV1 | BAV56295 | *Narnaviridae* | *Narnavirus* |
| *Botrytis cinerea mitovirus* 4 | BcMV4 | CEZ26303 | *Narnaviridae* | *Mitovirus* |
| *Sclerotinia sclerotiorum mitovirus* 4 | SsMV4 | AGC24233 | *Narnaviridae* | *Mitovirus* |
| *Ophiostoma mitovirus* 4 | OpMV4 | CAB42652 | *Narnaviridae* | *Mitovirus* |
| *Thielaviopsis basicola mitovirus* | TbMV | AAZ99833 | *Narnaviridae* | *Mitovirus* |
| *Sclerotinia nivalis mitovirus* 2 | SnMV2 | ANJ77670 | *Narnaviridae* | *Mitovirus* |
| *Fusarium poae mitovirus* 1 | FpMV1 | BAV56289 | *Narnaviridae* | *Mitovirus* |
| *Fusarium poae mitovirus* 3 | FpMV3 | BAV56291 | *Narnaviridae* | *Mitovirus* |
| *Fusarium poae mitovirus* 2 | FpMV2 | BAV56290 | *Narnaviridae* | *Mitovirus* |
| *Alternaria arborescens mitovirus* 1 | AaMV1 | BAV53122 | *Narnaviridae* | *Mitovirus* |
| *Cronartium ribicola mitovirus* 1 | CrMV1 | AMQ67414 | *Narnaviridae* | *Mitovirus* |
| *Gremmeniella abietina* mitochondrial RNA virus S2 | GaMRV1 S2 | AAT48883 | *Narnaviridae* | *Mitovirus* |
| Ourmia melon virus | OuMV | ACF16360 | unassigned | *Ourmiavirus* |
| Epirus cherry virus | EcV | ACF16357 | unassigned | *Ourmiavirus* |
| Cassava virus C | CaV C | ACI03053 | unassigned | *Ourmiavirus* |
| Wenling narna-like virus 3 | WlNLV3 | APG77258 | unassigned | unassigned |
| Wenzhou narna-like virus 4 | WzNLV4 | APG77304 | unassigned | unassigned |
| Beihai narna-like virus 10 | BhNLV10 | APG77091 | unassigned | unassigned |
| Beihai narna-like virus 6 | BhNLV6 | APG77080 | unassigned | unassigned |
| *Enterobacteria* phage MS2 | EnP MS2 | CAA23991 | *Leviviridae* | *Levivirus* |
| *Enterobacteria* phage Hgal1 | EnP Hga11 | AFN37819 | *Leviviridae* | *Levivirus* |
| *Enterobacteria* phage GA | EnP GA | CAA27499 | *Leviviridae* | *Levivirus* |
| *Enterobacteria* phage C-1 INW-2012 | EnP C-1 | AFN37815 | *Leviviridae* | *Levivirus* |
| *Escherichia* virus FI | EsV FI | ABK60124 | *Leviviridae* | *Allolevivirus* |
| *Enterobacteri*a phage MX1 | EnP MX1 | AAC14701 | *Leviviridae* | *Allolevivirus* |
| *Enterobacteria* phage SP | EnP SP | CAA30375 | *Leviviridae* | *Allolevivirus* |

Note: ‘Polymycoviridae’ was a newly proposed family by Kotta-Loizou and Coutts^10^, but has not been approved by International Committee on Taxonomy of Viruses (ICTV) yet.

**Table S3** Peptide mass fingerprinting analysis of the protein encoded by Pm dsRNA4.

| Amino acid position | Calculated mass | Observed mass | ± delta | Amino acid sequence |
| --- | --- | --- | --- | --- |
| 19-34 | 1729.91 | 1729.89 | -0.0210 | ADLDQSVVDVILRLSS |
| 39-49 | 1264.39 | 1264.21 | -0.1822 | PDGIIDYCNRV |
| 52-66 | 1391.56 | 1391.48 | -0.0860 | DEPAPAVVAGGAKPL |
| 101-117 | 1957.20 | 1957.18 | -0.0217 | DPEEGRRIVHKAVADHQ |
| 115-129 | 1645.91 | 1645.89 | -0.0328 | DHQAKRGSPKPIVVN |
| 130-147 | 1583.70 | 1583.69 | -0.0171 | LAGLPSSRGGPKTGGDGG |
| 151-173 | 2533.85 | 2533.83 | -0.0215 | LSELMRRNQALAGAYAFVAEEHG |
| 192-201 | 989.15 | 989.09 | -0.0616 | GQNKACAVKA R |
| 244-258 | 1571.59 | 1571.58 | -0.0132 | APDEKPPSDESKGQS |

**Table S4** Changes of prochloraz EC_50_ values of *P. digitatum* strains when infected with mycovirus(es)

| Strains | Virus-free | Virus-infected | | |
| --- | --- | --- | --- | --- |
|  |  | PdV1 | PdPmV1+PdNLV1 | PdPGV1 |
| HS-F6 | 7.90±0.21a | 7.82±0.24a | 3.81±0.17b | 7.76±0.15a |
| HS-L17 | 5.94±0.16a | 6.02±0.11a | 4.10±0.24b | 5.83±0.31a |
| HS-K25 | 3.94±0.09a | 4.02±0.12a | 2.35±0.12b | 3.89±0.10a |
| HS-L8 | 1.05±0.09a | 1.09±0.14a | 0.69±0.05b | 1.01±0.11a |
| HS-E9 | 0.94±0.08a | 0.91±0.05a | 0.64±0.04b | 0.98±0.09a |
| HS-K11 | 0.93±0.05a | 0.89±0.07a | 0.67±0.06b | 0.85±0.04a |

Letters indicate statistical differences (*P* < 0.05).

**Table S5** Information on the proteins encoded by previously database-registered dsRNA2 and dsRNA3 of polymycoviruses used for sequence alignment in this work.

| Virus name | Abbreviation | Accession number | |
| --- | --- | --- | --- |
|  |  | dsRNA2-encoded-protein | dsRNA3-encoded-protein |
| *Aspergillus fumigatus* tetramycovirus-1 | AfuPmV-1 | CDP74619.1 | CDP74620.1 |
| *Botryosphaeria dothidea* RNA virus1 | BdRV1 | YP_009342447.1 | ALZ41796.1 |
| *Beauveria bassiana* polymycovirus-1 | BbPmV-1 | YP_009352876.1 | YP_009352877.1 |
| *Alternaria tenuissima* virus | AtV | ACL80752.1 |  |
| *Cladosporium cladosporioides* virus1 | CcV-1 | YP_009052471.1 | YP_009052472.1 |
| *Melampsora lini* virus | MlV |  | CAA45724.1 |
